# Integrating incompatible tandem photobiocatalysis in artificial cells enables metabolic modulation of natural cells

**DOI:** 10.1126/sciadv.adu4828

**Published:** 2025-07-04

**Authors:** Zhicheng Wang, Sharafudheen Pottanam Chali, Thao P. Doan-Nguyen, Seunghyeon Kim, Volker Mailänder, Shuai Jiang, Katharina Landfester

**Affiliations:** ^1^ Max Planck Institute for Polymer Research, Ackermannweg 10, 55128 Mainz, Germany.; ^2^ Dermatology Department, University Medicine Mainz, Langenbeckstr. 1, 55131 Mainz, Germany.; ^3^ Key Laboratory of Marine Drugs, Chinese Ministry of Education, School of Medicine and Pharmacy, Ocean University of China, Qingdao 266003, PR China.; ^4^ State Key Laboratory of Marine Food Processing and Safety Control, Ocean University of China, Qingdao 266404, PR China.; ^5^ Laboratory for Marine Drugs and Bioproducts, Qingdao Marine Science and Technology Center, Qingdao 266237, PR China.

## Abstract

Sustaining biological reactions in artificial cells is crucial for their practical integration into living systems, which relies on continuous cofactor supply. Although photocatalysis enables cofactor regeneration in synthetic biological systems, the generated reactive oxygen can deactivate enzymes. Here, we engineer photobiocatalytic artificial cells that modulate hepatocyte metabolism through alleviating alcohol-induced oxidative stress. These artificial cells feature nano-organelles that segregate incompatible modules: one for photocatalytic cofactor regeneration and another for biocatalytic alcohol metabolism. This spatial separation ensures sustainable cofactor provision and protects enzymes from oxidative damage. Co-compartmentalization of alcohol dehydrogenase and aldehyde dehydrogenase within a single nano-organelle enhances cascade reaction efficiency while inhibiting intermediate leakage. When cocultured with hepatocytes, these artificial cells demonstrate excellent biocompatibility and efficiently mitigate oxidative stress from alcohol metabolism. This work advances artificial cells from proof of concept to practical application in living systems. The successful connection of photocatalysis and enzymatic reactions broadens the range of strategies available for chemical synthesis, synthetic biology, and biomedical applications.

## Teaser

Artificial cells with segregated photobiocatalysis sustain alcohol metabolism and protect cells from alcohol-induced stress.

## INTRODUCTION

In this study, we created hierarchical artificial cells featuring customizable subcellular compartments, enabling the integration of incompatible photo- and biocatalytic modules in separate artificial nano-organelles. These “cells” mimic hepatocytes, efficiently modulating hepatocyte metabolism by alleviating oxidative stress induced by alcohol metabolism.

Recently, various cell-mimicking systems based on liposomes (*1*–*5*), polymersomes (*6*–*8*), hydrogels (*9*, *10*), or coacervates (*11*–*13*) have been developed to artificially replicate the structure and complex biological processes of natural cells. However, sustaining biological reactions in these systems poses considerable challenges, primarily due to the necessity of continuous cofactor supply, which is also the prerequisite for the sustained function of living cells. Cofactors such as NAD^+^ (β-nicotinamide adenine dinucleotide) and NADP^+^ (β-nicotinamide adenine dinucleotide phosphate) play pivotal roles in their oxidized forms, typically used by dehydrogenases to catalyze specific substrates (*14*–*16*). Despite advancements in enzymatic (*17*, *18*), chemical (*19*), chemoenzymatic (*20*), and electrochemical (*21*) cofactor regeneration systems, their application in living systems is hindered because of required complex components, biologically incompatible reaction conditions, and use of organic reagents (*22*). Visible light photocatalysis has emerged as a selective method to catalyze reactions under mild conditions, enabling a broad range of redox reactions beyond enzymatic capabilities and showing great potential for cofactor regeneration in synthetic biological systems (*23*–*26*). Coupling photocatalysis with biocatalysis opens up possibilities for traditionally challenging reaction processes (*27*–*32*). However, the generation of reactive oxygen species (ROS) during photocatalysis can deactivate enzymes, posing compatibility issues that impede broader adoption of photobiocatalysis (*33*). To address the enzyme denaturation resulting from ROS exposure, physical segregation of photocatalysts and enzymes within distinct subcellular compartments is crucial, which ensures the transformation of ROS into a harmless state before interacting with enzymes, leveraging the short lifetime of ROS (*24*, *25*).

**Fig. 1.**
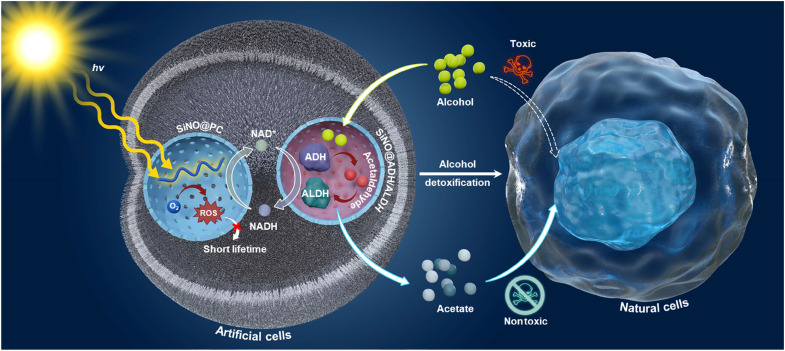
Schematic illustration of artificial cells modulating the metabolism of natural cells. The multicompartmentalized artificial cells integrating two incompatible modules, i.e., a photocatalytic cofactor regeneration system and a biocatalytic alcohol metabolism system, within distinct nano-organelles. A hydrophilic PC was confined within SiNOs to create the photocatalytic module (SiNO@PC). In parallel, ADH and ALDH were spatially coupled within SiNOs at their optimized ratio, forming the biocatalytic module (SiNO@ADH/ALDH). Last, the two modules are encapsulated in pGUVs via bottom-up assembly to form functional artificial cells, in which the photocatalytic module regenerates the NAD^+^ to sustain the biocatalytic module. By segregating photocatalysts and enzymes into different SiNOs, enzymes are shielded from oxidative damage by photocatalysis, attributed to the short lifetime of ROS. Co-compartmentalization of ADH and ALDH within a single nano-organelle enhances the overall efficiency of cascade reaction by accelerating the decomposition of intermediate product acetaldehyde within the confined space and meanwhile inhibits intermediate leakage. These artificial cells modulate natural cell metabolism for alcohol detoxification.

Here, we engineered artificial cells featuring subcellularly confined segregation of two incompatible modules, i.e., a photocatalytic cofactor regeneration system and a biocatalytic alcohol metabolism system, within distinct artificial nano-organelles (Fig. 1). Specifically, a hydrophilic photocatalytic polymer (PC) was synthesized and confined within silica nano-organelles (SiNOs) to create the photocatalytic module (SiNO@PC). In parallel, alcohol dehydrogenase (ADH) and aldehyde dehydrogenase (ALDH) were spatially coupled within SiNOs at their optimized ratio, forming the biocatalytic module (SiNO@ADH/ALDH). The semipermeable shell of the SiNOs facilitates efficient substrate and product transportation across it. Last, the two modules were encapsulated in polymeric giant unilamellar vesicles (pGUVs), also known as giant polymersomes, via bottom-up assembly to obtain functional artificial cells, in which the photocatalytic module regenerates the NAD^+^ to sustain the biocatalytic module. Moreover, by segregating photocatalysts and enzymes into different SiNOs, enzymes are shielded from oxidative damage by photocatalysis, attributed to the short lifetime of ROS. Co-compartmentalization of ADH and ALDH within the same SiNO enhances overall cascade reaction efficiency by accelerating the decomposition of intermediate product acetaldehyde within the SiNO cavity. This hierarchical compartmentalization strategy enables subcellular intercommunication between nano-organelles without mutual interference. When cocultured with living cells, the photobiocatalytic artificial cells exhibited excellent biocompatibility and efficiently reduced oxidative stress in hepatocytes upon exposure to alcohol and acetaldehyde in vitro. This work demonstrates the successful integration of originally incompatible photoenzymatic catalysis processes for cell metabolic modulation, paving the way for the design of new-to-nature chemical transformations and expanding possibilities in chemical synthesis and synthetic biology.

## RESULTS

### Synthesis of PC for cofactor regeneration

Conjugated polymer photocatalysts emerge as promising candidates for various catalytic applications owing to their robust structures, favorable biocompatibility, customizable designs, and high catalytic efficiency (*34*). By precisely adjusting their electronic, optical, and chemical properties via doping, molecular engineering, and surface functionalization, these polymers can be finely tailored to address specific photocatalytic demands (*35*). Here, we synthesized fluorene-and benzothiadiazole-based linear conjugated polymers, featuring visible light responsiveness, good biocompatibility, hydrophilicity, and excellent photocatalytic performance, for catalyzing the regeneration of cofactor NAD^+^. The poly[(9,9-bis(6-N,N-diethyl-N-methylammoniumhexyl)fluorene)-*alt*-benzothiadiazole] (P-BT-QA) PC was synthesized in three steps. First, bromide-based conjugated polymer poly[(9,9-bis(6-bromohexyl)fluorene)-*alt*-benzothiadiazole] (P-BT-Br) was synthesized via the Suzuki coupling reaction (fig. S1). Subsequently, the bromide of P-BT-Br was substituted with diethylamine to form a tertiary amine, forming poly[(9,9-bis(6-N,N-diethylaminehexyl)fluorene)-*alt*-benzothiadiazole] (P-BT-DEA) (fig. S2). Last, hydrophilic ionized P-BT-QA was obtained upon reaction of P-BT-DEA with iodomethane (fig. S3). The formation of P-BT-DEA was confirmed through Fourier transform infrared spectroscopy (fig. S4). The peak corresponding to the C-Br stretching vibration (556 cm^−1^) disappeared in the spectra of P-BT-DEA and P-BT-QA, indicating successful substitution of bromide with amines. Peaks observed at 1190, 1394, and 2965 cm^−1^ were attributed to C-N stretching, N-CH deformation, and N-CH_3_ stretching vibrations, respectively, in both P-BT-DEA and P-BT-QA. The presence of N-CH_2_ stretching vibrations at 2795 cm^−1^ in P-BT-DEA, consistent with previous findings (*36*), was absent in P-BT-QA, indicating successful formation of quaternary ammonium on P-BT-QA. Furthermore, ultraviolet-visible (UV-Vis) spectrum of P-BT-QA in water exhibited absorption ranging from 400 to 540 nm in the visible spectrum, with the peak absorption at 460 nm, demonstrating its potential for photocatalysis under visible light (fig. S5).

### Engineering of photocatalytic nano-organelles for cofactor regeneration

**Fig. 2.**
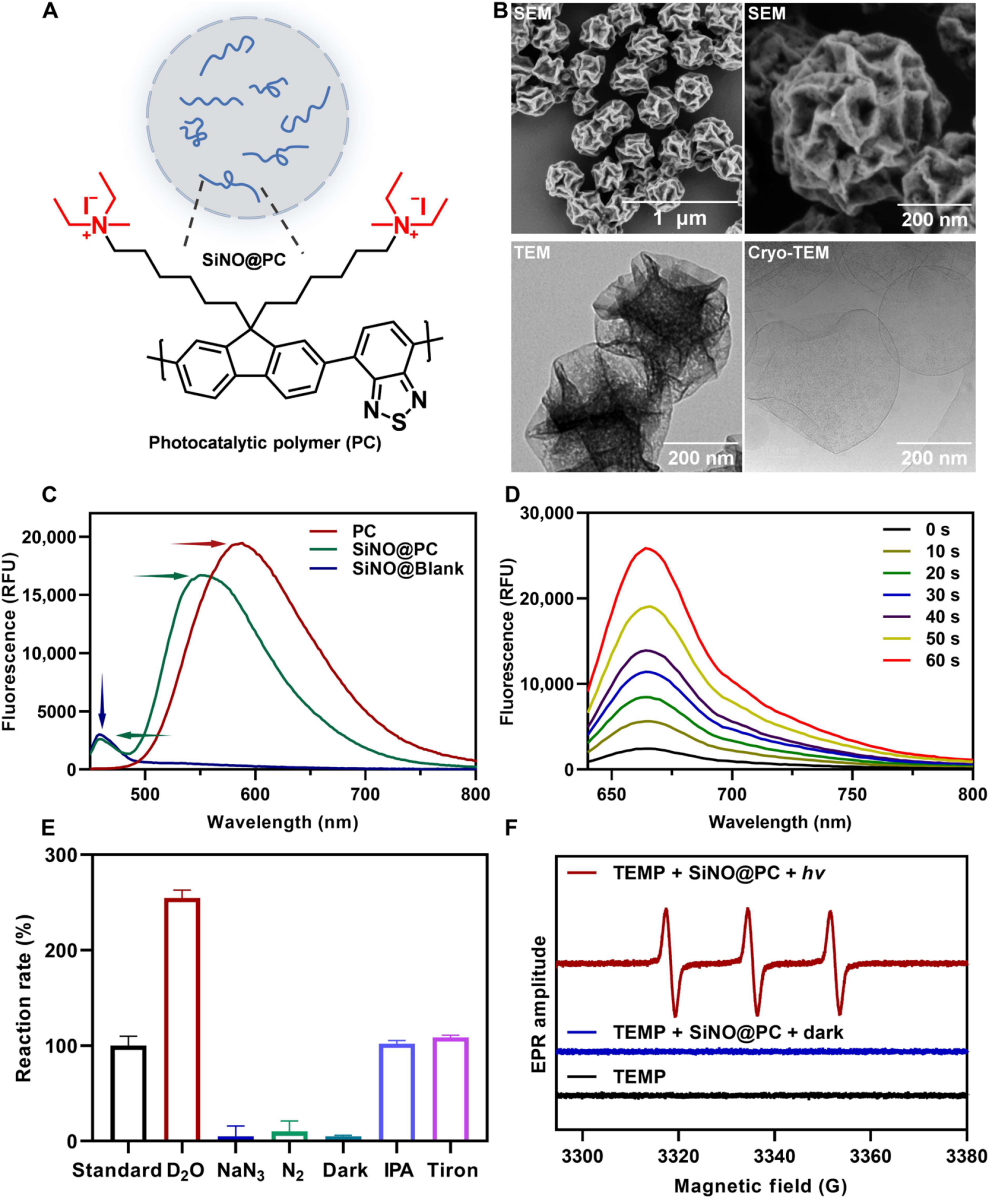
Construction and characterization of photocatalytic nano-organelles for cofactor regeneration. (**A**) Schematic illustration of photocatalytic nano-organelles comprising PC encapsulated in silica nanocapsules (SiNO@PC). (**B**) SEM, TEM, and cryo-TEM images of SiNO@PC. (**C**) Comparison of the photoluminescence (λ_ex_ = 323 nm) of PC, SiNO@PC, and SiNO@Blank. RFU, relative fluorescent units. (**D**) Change in fluorescence spectra of ROS Brite 670 (λ_ex_ = 600 nm) upon treatment with SiNO@PC under light irradiation over a period of 60 s. The ROS Brite 670 is an ROS-responsive probe that can be excited from nonfluorescence to a fluorescent state (λ_em_ = 670 nm) by ROS oxidation. (**E**) Effect of reaction conditions and scavengers on NAD^+^ regeneration by SiNO@PC. The experiments were conducted using D_2_O as a solvent, N_2_ atmosphere as the reaction environment, or a dark condition. NaN_3_, IPA, and Tiron were introduced as scavengers for quenching ^1^O_2_, ^•^OH, and ^•^O_2_^−^, respectively. (**F**) EPR spin trapping spectra of TEMP-^1^O_2_ generated under different conditions.

The hydrophilic PC was directly encapsulated within the aqueous core of silica nanocapsules during their formation in an inverse miniemulsion (Fig. 2A and fig. S6) (*37*). Initially, PC was dissolved in water to create the aqueous phase, which was transformed into nanodroplets dispersed in cyclohexane through microfluidization processing. To selectively form a silica shell around nanodroplets, we introduced an aminosilane into the silica precursors, which acts as a catalyst as well as an amphiphilic anchor that electrostatically assembles with negatively charged hydrolyzed alkoxysilanes at the water-oil interface. Consequently, the sol-gel reaction was confined at the nanodroplet surface, resulting in the formation of aqueous core-mesoporous shell nanocapsules. These nanocapsules have a spacious interior for payload storage and a semipermeable shell that entraps the PC while allowing the diffusion of reaction components. Last, the nanocapsules prepared in cyclohexane were transferred to an aqueous medium with the aid of a poly(ethylene glycol)–based surfactant Lutensol AT 50 to enhance their stability in biological environments.

Dynamic light scattering (DLS) studies confirmed the formation of SiNOs with a diameter of approximately 300 nm and a narrow size distribution [polydispersity index (PDI) = 0.055] (fig. S7). Scanning electron microscopy (SEM) and transmission electron microscopy (TEM; Fig. 2B) images revealed nanoparticles with a spherical morphology and uniform sizes consistent with the DLS results. The small pore size (5 nm), determined by Brunauer-Emmett-Teller analysis (fig. S8), facilitated efficient trapping of the PC inside the SiNOs. In addition, cryo–transmission electron microscopy (cryo-TEM) image revealed a distinct hollow capsule structure with an ultrathin shell (Fig. 2B), underscoring the potential of SiNOs as an optimal system for nano-organelles. To confirm the successful encapsulation of PC within SiNOs, we measured the photoluminescence spectra (Fig. 2C). The maximum emission wavelengths of PC and SiNO@Blank (empty SiNOs) were observed at 583 and 458 nm, respectively. The purified SiNO@PC exhibited two peaks corresponding to PC and SiNO@Blank, confirming successful encapsulation of PC inside the SiNOs. The loading content of PC in SiNOs was evaluated using thermogravimetric analysis (fig. S9). The SiNO@PC was heated up to 500°C under nitrogen gas. Compared to SiNO@Blank, the SiNO@PC group showed a 2.01% additional weight loss at 250°C, indicating effective encapsulation of PC in SiNOs. In addition, the negligible variation in particle size, zeta potential, and residual PC loading over time indicates good stability of SiNO@PC (figs. S10 and S11). Optical properties of PC and SiNO@PC were studied using UV-Vis diffuse reflectance spectroscopy (fig. S12). The results revealed that PC and SiNO@PC have broad absorption bands up to 700 and 510 nm in the visible range, respectively (fig. S12, A and B). Through calculation with the Kubelka-Munk function, the optical bandgaps of PC and SiNO@PC were determined to be 2.16 and 2.47 eV, respectively (fig. S12, D and E). To further characterize the electronic properties, specifically the lowest unoccupied molecular orbital (LUMO) and the highest occupied molecular orbital (HOMO), we used cyclic voltammetry (CV) measurements. According to the result of LUMO location at −0.79 V versus saturated calomel electrode (SCE) from CV results, the HOMO of PC was calculated to be 1.37 V versus SCE (fig. S12, G and H). The HOMO/LUMO of SiNO@PC had a similar value (1.76/−0.71 V versus SCE) to that of PC, indicating that the electronic structure of PC was preserved upon encapsulation in the SiNOs.

To verify the capability of SiNO@PC in regenerating NAD^+^ from the reduced form NADH, we recorded the ^1^H NMR and UV-Vis spectra following irradiation with 460-nm blue light (fig. S13). The characteristic chemical shift of adenine protons transitioned from δ = 8.24 and 8.49 parts per million (ppm) in NADH to δ = 8.18 and 8.44 ppm in NAD^+^, with dehydrogenation of the nicotinamide ring, the peak of nicotinamide signals in NADH disappearing at δ = 6.94 and in NAD^+^ appearing at δ = 8.83, 9.15, and 9.34 ppm. Meanwhile, NADH absorbance appeared at 340 nm, validating the conversion of NADH to NAD^+^ catalyzed by SiNO@PC. The SiNO@PC exhibited catalytic efficiency only slightly lower than that of free PC, which may be attributed to the localized enrichment and good hydrophilicity of PC in nanocapsules (fig. S14). ROS generation by SiNO@PC and SiNO@Blank was assessed using the ROS Brite 670 probe, which produces fluorescence at 670 nm upon ROS oxidation. Under 5 mW/cm^2^ blue-light irradiation, the fluorescence intensity of SiNO@PC showed a continuous increase (Fig. 2D), while the control group SiNO@Blank exhibited no change in fluorescence intensity (fig. S15). To identify the types of ROS generated by SiNO@PC, we explored various reaction conditions, including the use of D_2_O as a solvent, a nitrogen atmosphere, and dark conditions. D_2_O was used to replace H_2_O due to the longer lifetime of ^1^O_2_ in D_2_O (*38*). In addition, three scavengers [sodium azide (NaN_3_), isopropanol (IPA), and Tiron] were used for quenching ^1^O_2_, ^•^OH, and ^•^O_2_^−^, respectively. Control reactions in the presence of scavengers revealed that ^1^O_2_, rather than ^•^OH or ^•^O_2_^−^, is the dominant species driving the oxidation of NADH (Fig. 2E). To further confirm ^1^O_2_ generation, we used 9,10-anthracenediyl-bis(methylene)dimalonic acid (ABDA) as both an ^1^O_2_ indicator and a competitor. In the ABDA + NADH group, oxidation rates of both ABDA and NADH were markedly reduced compared to the groups containing ABDA or NADH alone, confirming the critical role of ^1^O_2_ (fig. S16). Electron paramagnetic resonance (EPR) spectroscopy further confirmed the generation of ^1^O_2_ (Fig. 2F). Given the longer life span and stability of H_2_O_2_, we hypothesized that it might form as a downstream product of ROS decay. To test this, we conducted a horseradish peroxidase–coupled 2,2′-azino-bis(3-ethylbenzothiazoline-6-sulfonic acid) diammonium salt oxidation assay to quantify H_2_O_2_ formation. However, no detectable H_2_O_2_ was observed (fig. S17), suggesting that ^1^O_2_ likely reverted to its ground state (triplet oxygen, O_2_) following the oxidation of NADH. Therefore, the inferred mechanism of PC-mediated NAD^+^ regeneration involves three reactions (fig. S18).

### Engineering of biocatalytic nano-organelles co-compartmentalizing cascade enzymes for alcohol metabolism

**Fig. 3.**
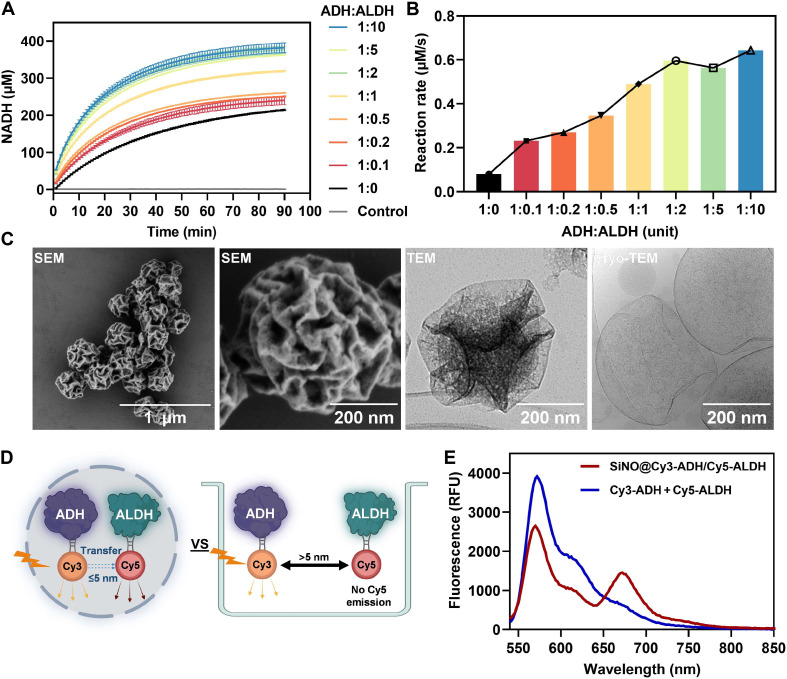
Engineering of biocatalytic nano-organelles with co-compartmentalized cascade enzymes. (**A**) Comparison of reaction rates for NADH generation using free enzymes with varying ADH/ALDH activity unit ratios. (**B**) Initial reaction rate of NADH generation in the first minute. (**C**) SEM, TEM, and cryo-TEM images of SiNO@ADH/ALDH. (**D**) Schematic illustration of FRET effect in SiNO@Cy3-ADH/Cy5-ALDH and mixture of Cy3-ADH and Cy5-ALDH in solution. ADH and ALDH were labeled with fluorophores Cy3 and Cy5, respectively. (**E**) Fluorescence spectra (λ_ex_ = 500 nm) of SiNO@Cy3-ADH/Cy5-ALDH and mixture of Cy3-ADH and Cy5-ALDH in solution.

During alcohol metabolism, the ethanol molecules are initially degraded by ADH to form acetaldehyde, a toxic compound further metabolized to acetic acid by ALDH. NAD^+^ serves as a crucial cofactor in both steps for substrate dehydrogenation. In liver cells, ADH and ALDH reside in separate compartments: ADH in the cytoplasm and ALDH in the mitochondria. This distinct subcellular compartmentalization reflects a regulated spatial organization that facilitates efficient ethanol metabolism, representing a refined outcome of mammalian evolution. However, in artificial systems, this spatial segregation may diminish the efficiency of cascade reaction and exacerbate the side effects arising from acetaldehyde release due to increased diffusion barriers. Hence, we developed a co-compartmentalization strategy to enhance the close proximity of ADH and ALDH while inhibiting the leakage of toxic intermediate acetaldehyde, aiming to shield liver cells from oxidative stress damage and mitigate associated side effects (*39*). To this aim, we first optimized the ratio of both enzymes to maximize cascade reaction efficiency. As illustrated by the overall generation rate of product NADH, the initial rate, acetaldehyde accumulation, and acetate production (Fig. 3, A and B, and fig. S19), the highest activity was achieved when the activity unit ratio of ADH to ALDH was 1:2, 1:5, and 1:10. Considering the low solubility and activity of ALDH [lyophilizate (2.4 U/mg), Roche, Germany] compared to ADH [lyophilizate (400 U/mg), Sigma-Aldrich, Germany] and to prevent overcrowding inside SiNOs, the activity unit ratio 1:2 (U_ADH_:U_ALDH_) was chosen for encapsulation in the SiNOs. Following the same protocol as the preparation of SiNO@PC, the enzymes ADH and ALDH were co-encapsulated in SiNOs to prepare SiNO@ADH/ALDH, which exhibited similar morphology and size to SiNO@PC (Fig. 3C). To confirm the co-encapsulation of enzymes in close proximity, we conducted a Förster resonance energy transfer (FRET) assay using a fluorescent probe pair to separately label the enzymes (Cy3-ADH and Cy5-ALDH). When the distance between Cy3 (donor) and Cy5 (acceptor) is below 5 nm, energy is transferred from donor to acceptor because the emission spectrum of Cy3 overlaps with the excitation spectrum of Cy5 (Fig. 3D). Compared to free enzymes (Cy3-ADH and Cy5-ALDH) in solution, SiNO@Cy3-ADH/Cy5-ALDH exhibited both Cy3 and Cy5 fluorescence under 500 nm excitation, with maximum emission wavelengths at 570 and 670 nm, respectively, indicating close proximity between the fluorophores (Fig. 3E). The short distance between ADH and ALDH favors efficient cascade reactions and elimination of intermediate acetaldehyde. The encapsulated amount of ADH and ALDH was quantified using ultrafiltration and measured with bicinchoninic acid (BCA) protein assay. The enzymes were almost completely segregated within the concentrates, with neglected amount detected in the ultrafiltrates, indicating a high encapsulation efficiency of enzymes through this in situ encapsulation approach (fig. S20).

### Tandem photobiocatalysis for collaborative alcohol metabolism

Despite advancements in alcohol antidotes, such as enzyme-encapsulated nanocapsules (*40*, *41*), silk enzymatic nanospheres (*42*), and metal organic framework–based nanoreactors (*39*), challenges persist because of overconsumption of coenzyme NAD^+^ and the generation of toxic intermediate products such as H_2_O_2_ and acetaldehyde. Therefore, addressing NAD^+^ regeneration and preventing the generation or release of toxic intermediate products are paramount in developing effective alcohol antidotes.

**Fig. 4.**
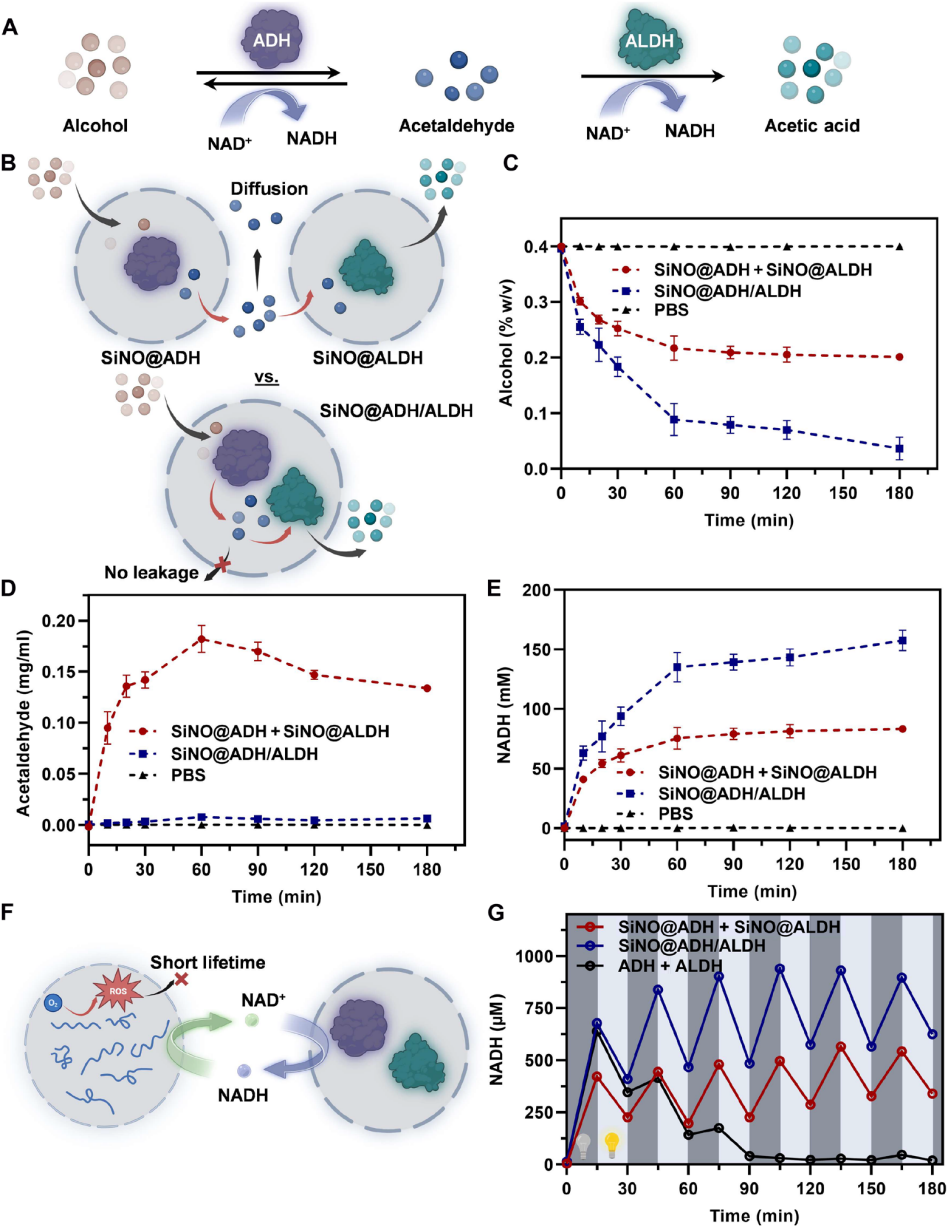
Confinement effect of enzymatic cascade reaction and combination of incompatible photobiocatalysis. (**A**) Cascade reaction scheme illustrating alcohol metabolism by ADH and ALDH. (**B**) Comparative scheme depicting the confinement effect of enzymatic cascade reaction for SiNO@ADH + SiNO@ALDH and SiNO@ADH/ALDH. (**C**) Variation in alcohol degradation, (**D**) accumulation of acetaldehyde, and (**E**) generation of NADH following incubation with SiNO@ADH + SiNO@ALDH or SiNO@ADH/ALDH. Data are presented as the means ± SD for *n* = 3 independent samples. PBS, phosphate-buffered saline. (**F**) Schematic illustration of the combination of photocatalytic nano-organelles SiNO@PC and biocatalytic cascade nano-organelles SiNO@ADH/ALDH for the NADH/NAD^+^ cycle. (**G**) Concentration of NADH generated from combinations of SiNO@PC with SiNO@ADH + SiNO@ALDH, SiNO@ADH/ALDH, or free enzymes ADH + ALDH, upon blue light off-on alternation. Dark and light colors represent blue light off and on, respectively.

To elucidate the advantage of co-encapsulating ADH and ALDH in SiNOs, we assessed alcohol degradation, acetaldehyde accumulation, NADH generation, and acetate production using alcohol solutions to mimic acute intoxication (Fig. 4, A and B) (*43*). SiNO@ADH/ALDH and SiNO@ADH + SiNO@ALDH were tested, and the results revealed that co-encapsulation in SiNO@ADH/ALDH led to 95% alcohol degradation within 3 hours, whereas only 50% degradation was observed with individually encapsulated enzymes (Fig. 4C). Moreover, negligible acetaldehyde accumulation was observed in SiNO@ADH/ALDH, in contrast to notable accumulation of acetaldehyde in SiNO@ADH + SiNO@ALDH due to intermediate leakage (Fig. 4D and fig. S21). In addition, SiNO@ADH/ALDH exhibited higher concentrations of NADH (Fig. 4E and fig. S22) and acetate (fig. S23) compared to the individual catalytic system, indicating improved cascade reaction efficiency and highlighting its potential for alcohol intoxication therapy.

However, both enzymes require the cofactor NAD^+^ for the dehydrogenation of substrates (*44*). Overconsumption of NAD^+^ can lead to reduced enzyme activity and the accumulation of toxic alcohol and acetaldehyde (*45*, *46*). Moreover, excessive NAD^+^ depletion could result in dysregulation of cellular energy homeostasis, causing potential hepatocyte lesions (*45*). Therefore, NAD^+^ regeneration is crucial for stabilizing enzyme activity and maintaining intracellular homeostasis. For this purpose, we constructed a cofactor regeneration system using photocatalytic nano-organelles (SiNO@PC) to regenerate NAD^+^ for enzymatic reactions (Fig. 4F). Quantitative ^1^H NMR analysis validated the specificity of SiNO@PC for NAD^+^ regeneration in the presence of alcohol (fig. S24). Specifically, the mixture of NADH and alcohol was subjected to SiNO@PC, and their residual percentages were calculated through peak integration using dimethyl sulfone as an internal standard. The integral values of unchanged alcohol and reduced NADH indicated the specificity of SiNO@PC for NAD^+^ regeneration.

Subsequently, the combination of photocatalytic and biocatalytic nano-organelles for the continuous cofactor regeneration and alcohol metabolism was determined (Fig. 4G). To initiate the photobiocatalytic cascade reaction, we added NAD^+^ and alcohol under dark conditions. The absorbance changes of NADH at 340 nm were monitored in 15-min intervals. After 15 min of reaction, blue light was applied, and the reaction mixture was irradiated for an additional 15 min to catalyze NADH oxidation. During this period, the concentration of NADH decreased because of the photocatalytic conversion of NADH back to NAD^+^. Multiple cycles of blue light on-off were conducted to demonstrate the successful cooperation of the combinatorial photoenzyme catalysis nano-organelles. Both the SiNO@ADH/ALDH and the SiNO@ADH + SiNO@ALDH exhibited fluctuation in NADH concentration corresponding to blue-light switching cycles, and co-encapsulated enzymes exhibited markedly higher reaction rates than separately encapsulated enzymes. Moreover, enzymes from both the co-encapsulated group and the separately encapsulated group were able to maintain enzymatic activity within six cycles. In contrast, the group of free enzymes (ADH + ALDH) nearly lost all enzymatic activity after two photocatalysis cycles due to direct exposure to ROS from SiNO@PC (Fig. 4G). Relative enzyme activity of ADH or ALDH after treatment with various light intensities or mixing with SiNO@PC under different conditions further confirmed enzymatic denaturation induced by ROS (fig. S25). This result underscores the importance of enzyme compartmentalization within the SiNOs to shield them from ROS damage by taking advantage of the short lifetime of ROS and limited diffusion distance (*38*). In addition, the interparticle spacing (IPS) relative to the volume fraction of particles in the colloidal system, calculated from the Riman model (*47*), revealed that even at a high particle concentration of 60 wt %, the spacing between SiNOs exceeded 10 nm (fig. S26).

### Photobiocatalytic artificial cells with hierarchical internal compartmentalization for alcohol metabolism

**Fig. 5.**
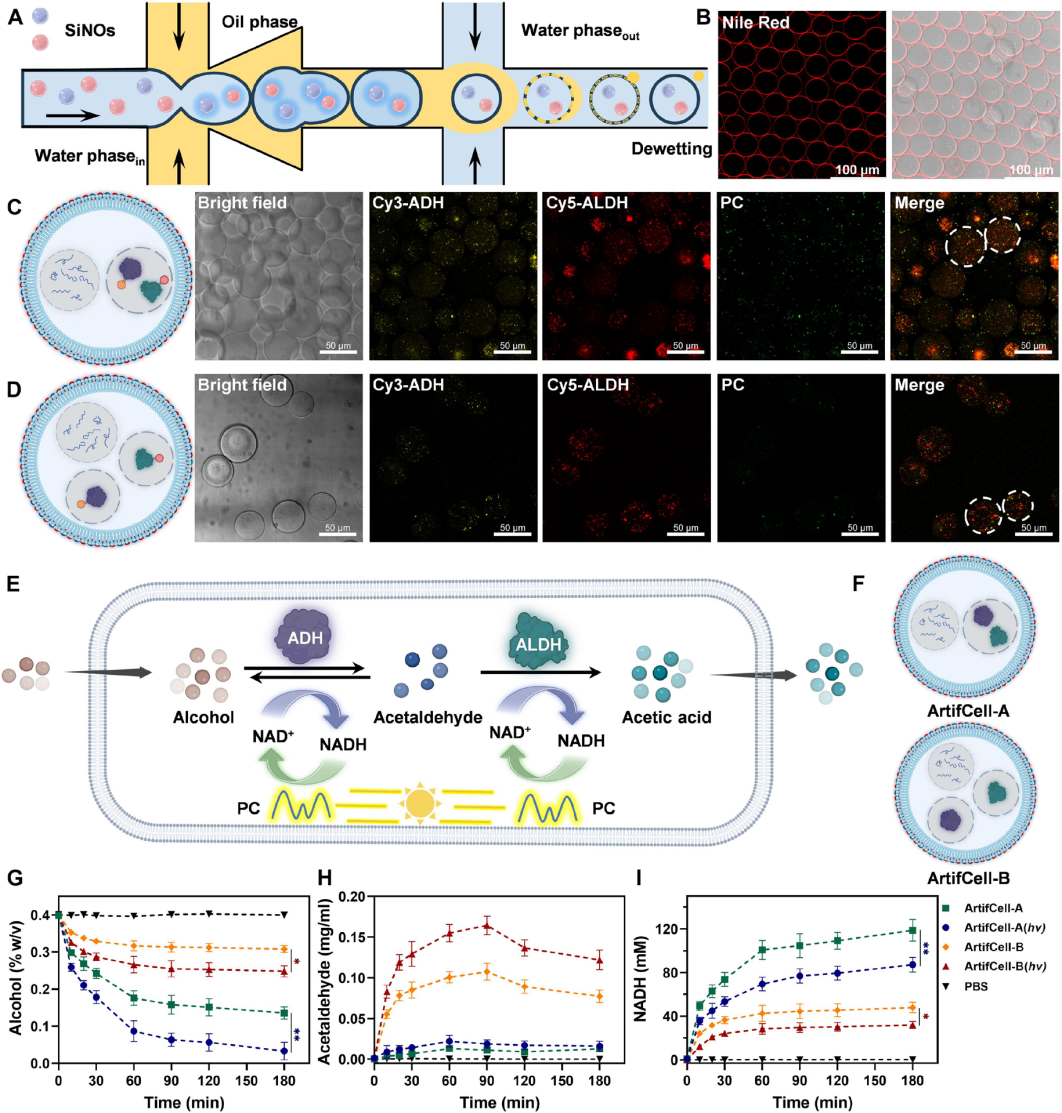
Artificial cells with photobiocatalytic nano-organelles for alcohol metabolism. (**A**) Schematic illustration of the formation of SiNO-encapsulated pGUVs using a microfluidic platform. (**B**) CLSM images of pGUVs with Nile Red–stained membranes. Scale bars, 100 μm. (**C**) CLSM images of pGUVs containing SiNO@PC and SiNO@Cy3-ADH/Cy5-ALDH. (**D**) CLSM images of pGUVs containing SiNO@PC, SiNO@Cy3-ADH, and SiNO@Cy5-ALDH. For (C) and (D): yellow: Cy3-ADH; red: Cy5-ADH; and green: PC. Scale bars, 50 μm. (**E**) Scheme illustrating the dynamic interplay between alcohol degradation by enzyme cascade reactions and NAD^+^ regeneration via photocatalysis within the pGUVs. (**F**) Scheme of photobiocatalytic artificial cells containing SiNO@ADH/ALDH (ArtifCell-A) or SiNO@ADH + SiNO@ALDH (ArtifCell-B), together with photocatalytic nano-organelles. Concentrations of alcohol (**G**), acetaldehyde (**H**), and NADH (**I**) following incubation with ArtifCell-A, ArtifCell-A(*hv*), ArtifCell-B, or ArtifCell-B(*hv*); *hv* = 460-nm blue light, 5 mW/cm^2^. The data are presented as the means ± SD for *n* = 3 independent samples. *P < 0.05; **P < 0.01.

To achieve efficient enzymatic cascade reactions and photocatalytic regeneration of cofactors, we constructed pGUV-based artificial cells using microfluidics (Fig. 5A and movie S1). The pGUVs are composed of amphiphilic copolymers PB_22_-*b*-PEO_14_ to simulate cellular membranes (*48*). The flowing phases in the fluid channel include an inner aqueous phase containing SiNOs, a middle oil phase dissolving the copolymer PB_22_-*b*-PEO_14_, and an outer aqueous phase with sodium chloride (figs. S27 and S28). Initially, at the first junction, the inner aqueous phase was dispersed in the middle oil phase, forming a water-in-oil emulsion. Subsequently, as this emulsion passed through the second junction, water-in-oil-in-water emulsions were formed. Following a dewetting transition, excess oil on the pGUV membrane departed, and SiNO-encapsulated pGUVs, namely, pGUV@SiNO, were obtained (Fig. 5B). Confocal laser scanning microscopy (CLSM) was used to visualize the distribution of SiNOs in artificial cells, using SiNOs containing PC, Cy3-labeled ADH, or Cy5-labeled ALDH. As shown in Fig. 5C, the co-encapsulated system SiNO@Cy3-ADH/Cy5-ALDH exhibited overlapped fluorescence of Cy3 and Cy5 inside artificial cells, along with separate fluorescence signal of the photocatalyst from SiNO@PC, indicating successful co-encapsulation of both SiNOs in a single pGUV. In comparison, the pGUV samples containing SiNO@Cy3-ADH + SiNO@Cy5-ALDH showed isolated fluorescence from Cy3 and Cy5, as well as separated signal from SiNO@PC (Fig. 5D). Furthermore, the comparable pGUV densities in Fig. 5C (3.17 × 10^6^ pGUVs/ml) and Fig. 5D (3.09 × 10^6^ pGUVs/ml) suggested the overall identical concentration. Quantitative analysis of the loading in pGUVs further demonstrated that SiNOs carrying PC or enzymes were co-encapsulated in pGUVs in a controllable manner (fig. S29). According to theoretical calculations, the average PC, ADH, and ALDH in each pGUV are approximately 9.09 × 10^−4^, 5.96 × 10^−4^, and 7.96 × 10^−2^ μg, respectively.

After confirming the hierarchical cell-like structure of artificial cells, collaboration between cofactor regenerating photocatalytic nano-organelles and biocatalytic nano-organelles within the pGUVs was further investigated. Upon passing through a membrane of pGUVs, the alcohol was first catalyzed by ADH to form an intermediate acetaldehyde, which was then oxidized by ALDH to produce acetic acid. Concurrently, the produced cofactor NADH from enzymatic reactions was oxidized to NAD^+^ by the photocatalyst under light irradiation (Fig. 5E). The efficacy of alcohol degradation, acetaldehyde accumulation, and NADH generation by (SiNO@PC + SiNO@ADH/ALDH)–encapsulated pGUVs (ArtifCell-A) and (SiNO@PC + SiNO@ADH + SiNO@ALDH)–encapsulated pGUVs (ArtifCell-B) was determined (Fig. 5F). Upon mixing alcohol solution with the artificial cells, a reduction in alcohol concentration was observed in both ArtifCell-A and ArtifCell-B systems (Fig. 5G). The trend of alcohol degradation by artificial cells mirrored that of the nano-organelles, with ArtifCell-A and ArtifCell-A(*hv*) showing less residual alcohol compared to ArtifCell-B and ArtifCell-B(*hv*). This highlights the role of close proximity and spatial nanoconfinement in enhancing the cascade reactions. Notably, ArtifCell-A(*hv*) demonstrated superior alcohol degradation compared to its nonirradiated counterpart, indicating efficient NAD^+^ regeneration within the artificial cell via its integrated photocatalytic nano-organelles. Although ArtifCell-B(*hv*) was also equipped with the same NAD^+^ regeneration module, the efficiency of alcohol degradation was hindered because of the separate encapsulation of ADH and ALDH. Consequently, a higher concentration of acetaldehyde was detected in the ArtifCell-B and ArtifCell-B(*hv*) groups due to outward diffusion of acetaldehyde from the ADH nano-organelles (Fig. 5H). In contrast, the produced acetaldehyde was immediately consumed when ADH and ALDH were co-encapsulated in the same nano-organelle. Moreover, the lower concentration of NADH and the higher level of acetate production in the ArtifCell-A(*hv*) group compared to ArtifCell-A suggest that the incorporation of a photocatalytic module enhanced the regenerative conversion of NADH to NAD^+^, thereby accelerating the overall reaction rate (Fig. 5I and fig. S30).

### Alcohol detoxification by photobiocatalytic artificial cells as hepatocyte-mimics

**Fig. 6.**
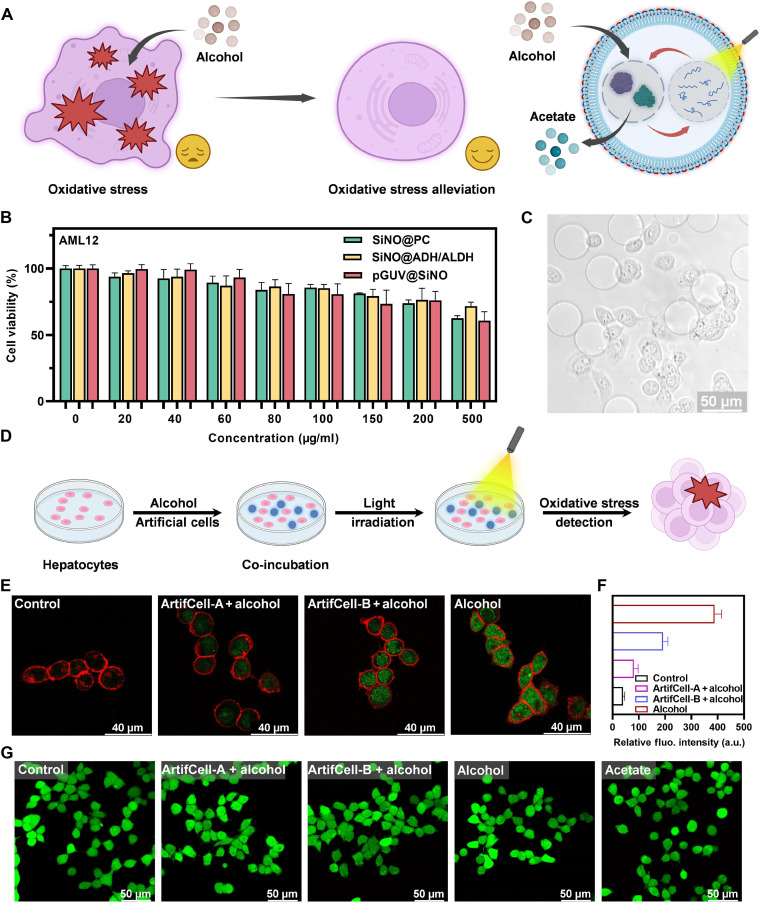
Photobiocatalytic artificial cells for alcohol detoxification. (**A**) Schematic depiction illustrating the role of artificial cells in alleviating oxidative stress induced by alcohol in living cells. (**B**) Viability of AML12 hepatocytes following incubation with SiNO@PC, SiNO@ADH/ALDH, and pGUV@SiNO at various concentrations for 24 hours. Data are presented as the means ± SD for *n* = 3 independent samples. (**C**) Bright-field image showing co-incubated AML12 hepatocytes and pGUVs. Scale bar, 100 μm. (**D**) Schematic illustration of co-incubation of AML12 hepatocytes and artificial cells for evaluating alcohol-induced intracellular oxidative stress. (**E**) CLSM images showing the oxidative stress level in AML12 hepatocytes after treatment with PBS, ArtifCell-A + alcohol, ArtifCell-B + alcohol, or alcohol. The alcohol-treated group (800 mM) was designated as the positive control, while the PBS-treated group served as the negative control. Intracellular ROS was detected with CellROX Green reagent, and cell membrane was stained with CellMask Deep Red. Scale bars, 40 μm. (**F**) Relative fluorescence intensity of ROS in AML12 hepatocytes was determined using a microplate reader. Data are presented as the means ± SD for *n* = 3 independent samples. The relative fluorescence intensity is expressed in arbitrary units (a.u.). (**G**) CLSM images of AML12 cell membrane permeability and integrity after treatment with PBS, ArtifCell-A + alcohol, ArtifCell-B + alcohol, alcohol, or acetate (sodium salt). The PBS-, alcohol (800 mM)–, and acetate (800 mM)–treated groups served as controls. Cells were stained with Calcein-AM and propidium iodide (PI) solution for live-dead cell imaging. Green: Calcein-AM; red: PI. Scale bars, 50 μm.

The capability of artificial cells in protecting living cells against alcohol-induced oxidative stress was further explored (Fig. 6A). First, we assessed the cytotoxicity of nano-organelles and artificial cells by incubating them with alpha mouse liver 12 (AML12) cells, which express sufficient levels of ADH and ALDH for alcohol metabolism (*49*). High viability of hepatocytes was observed even at a high concentration of SiNOs or pGUV@SiNO (500 μg/ml), validating their excellent biocompatibility (Fig. 6B). Moreover, the cells showed good tolerance to varying concentrations of alcohol and sodium acetate, as well as different intensities of 460-nm blue light applied in the study (fig. S31).

Subsequently, we investigated the alcohol metabolic reprogramming capability of artificial cells by coculturing them with AML12 cells in the presence of alcohol (Fig. 6, C and D, and fig. S32). The AML12 cells were initially exposed to 800 mM alcohol to induce oxidative stress, followed by assessment of intracellular ROS expression levels using CellROX Green probes after cultivation with artificial cells. As shown in Fig. 6E, compared to positive control (alcohol only, without artificial cells), CLSM images of ArtifCell-A + alcohol–treated and ArtifCell-B + alcohol–treated hepatocytes showed considerably reduced ROS expression, demonstrating their exceptional capability for mitigating intracellular oxidative stress. However, ArtifCell-B + alcohol–treated hepatocytes exhibited higher fluorescence intensity than ArtifCell-A + alcohol–treated hepatocytes. This result indicated that lower catalytic efficiency of alcohol metabolism and insufficient consumption of intermediate acetaldehyde by ArtifCell-B, which may cause acetaldehyde leakage, lead to heightened oxidative stress of the cells. Moreover, quantitative fluorescence intensity measurement via a microplate reader revealed a comparable trend as the fluorescence intensity in CLSM images for ArtifCell-A and ArtifCell-B (Fig. 6F). Although the experimental extracellular concentrations of cofactor NAD(H) (millimolar level) exceed physiological levels of natural cells (micromolar level), the exogenous NAD(H) is unlikely to induce nonphysiological side effects in natural cells, such as oxidative stress, redox imbalance, or enzyme inhibition, because it is a negatively charged, hydrophilic molecule that does not readily cross intact cell membranes (*50*, *51*). Notably, the live-dead cell imaging assay demonstrated that the artificial cells did not exert any impact on the permeability or integrity of the cell membrane (Fig. 6G). The confirmation of in vitro biocompatibility and alcohol metabolism programming capability of the photobiocatalytic artificial cells marks a notable step toward further translation of artificial cell technology.

## DISCUSSION

In this work, we engineered multicompartmentalized artificial cells for metabolic modulation of natural cells, enabling precise integration of incompatible photocatalytic modules with biocatalytic modules through selective encapsulation of photocatalysts and enzymes within SiNOs. These “cells” comprise dual nano-organelle modules: one contains a hydrophilic PC for efficient cofactor NAD^+^ regeneration, while the other hosts cascade enzymes, ADH and ALDH at optimized ratios, for alcohol metabolism. Encapsulation within artificial cell membranes creates a confined environment, facilitating continuous degradation of substrate alcohol to final nontoxic products via efficiently consuming the intermediate acetaldehyde, while minimizing its accumulation and leakage. Demonstrating excellent biocompatibility with living cells, our artificial cells markedly reduced oxidative stress in AML12 hepatocytes exposed to alcohol and acetaldehyde. By achieving subcellular-level selective and quantitative compartmentalization, our work effectively bridges the gap between photocatalysis and biocatalysis in synthetic living systems, thus advancing the potential for real biological applications of synthetic cells and their integration with living organisms. Future efforts to optimize the photocatalytic performance of polymeric photocatalysts will focus on enhancing light-harvesting capabilities, incorporating redox mediators to accelerate electron transfer, and using dynamic light regulation to balance reactant and product fluxes. Concurrently, integrating photocatalysis with enzymatic reactions holds great promise for addressing challenges plaguing chemical synthesis and synthetic biology.

## MATERIALS AND METHODS

### Synthesis of PCs

P-BT-Br was synthesized according to our previous study (*52*). Briefly, 2,7-dibromo-9,9-bis(6-bromohexyl)fluorene (1 mmol, 1 equiv) and 2,1,3-benzothiadiazole-4,7-diboronic acid pinacol ester (1 mmol, 1 equiv) were taken in a Schlenk tube and dissolved in 6 ml of dry tetrahydrofuran (THF). Subsequently, tetrakistriphenylphosphinpalladium(0) (0.06 mmol, 6 mol %), XPhos (0.06 mmol, 6 mol %), and potassium carbonate aqueous solution (6.99 mmol) were added. The mixture was purged with N_2_ under rigorous stirring and was further reacted at 80°C for 18 hours under N_2_ atmosphere. After that, the product was precipitated by adding 100 ml of water, followed by extraction of the product by 100 ml of dichloromethane. The final product was concentrated under reduced pressure and purified with Soxhlet apparatus. *M*_n_: 7600; *M*_w_: 23,000; PDI: 3.02 (gel permeation chromatography); and ^1^H NMR (400 MHz, CDCl_3_, at 298 K): δ = 8.06 (8 H), 3.30 (4 H), 2.17 (4 H), 1.70 (4 H), 1.28 (8 H), 0.94, and 0.77 (4 H).

P-BT-DEA was synthesized through the reaction of diethylamine with P-BT-Br in dry THF. Under N_2_ purging, the mixture was reacted at 50°C for 24 hours. Afterward, the solution was filtered to remove insoluble ammonium bromide salt. Subsequently, the solvent and extra diethylamine were evaporated with a rotary evaporator. The product was purified by washing with water and acetone. ^1^H NMR (400 MHz, CD_2_Cl_2_, at 298 K): δ = 8.12 (8 H), 2.38 (8 H), 2.27 (4 H), 1.71 (4 H), 1.27, 1.18, and 0.9 (28 H).

For the synthesis of P-BT-QA, the obtained P-BT-DEA was dissolved in dichloromethane, and then, iodomethane was added to the solution dropwise. Sticky dark-colored P-BT-QA was obtained and washed repeatedly with water and ethanol. The red orange powder product was obtained after drying at 65°C under vacuum overnight. ^1^H NMR [400 MHz, dimethyl sulfoxide (DMSO)–*d*_6_, at 298 K]: δ = 8.25 (8 H), 3.18 (8 H), 3.05 (4 H), 2.83 (6 H), 1.46 (4 H), and 1.11 (28 H).

### Preparation of silica nanocapsule–based nano-organelles

Photocatalyst- or enzyme-loaded SiNOs were prepared by inverse (water-in-oil) miniemulsion polymerization technique. First, PC or enzymes (ADH and ALDH) were separately dissolved in aqueous solutions to form the aqueous phase for emulsion preparation. Specifically, the PC was dissolved in water to obtain PC solution (1.5 mg/ml), and the enzymes were dissolved in tris-HCl buffer (100 mM, pH 8.0) to obtain a solution containing ADH (150 U/ml) and ALDH (300 U/ml). Then, the aqueous phase solution was transferred to an oil phase solution [polyglycerol polyricinoleate (PGPR) surfactant (26.7 mg/ml) in cyclohexane] under stirring at 1000 rpm, followed by pre-emulsification with using a T25 Ultra-Turrax (10,000 rpm for 1 min). Afterward, the pre-emulsified solution was processed by a microfluidizer (LM10, Microfluidics Corporation) twice under 6000 psi (41.37 N/mm^2^), to obtain the final emulsion. A mixed solution of silica precursors (tetramethyl orthosilicate:3-aminopropyltriethoxysilane = 4.50:0.85, molar ratio) was added dropwise to the emulsion, which was further stirred at 1000 rpm and room temperature for 2 hours to allow the sol-gel reaction to finish. Afterward, the obtained SiNOs were washed three times with cyclohexane to remove excessive PGPR. The purified SiNO solution (0.6 ml) was slowly added to 6 ml of 0.3 wt % Lutensol AT 50 aqueous solution under cyclic shaking in a sonication bath for 5 min, obtaining a water-in-oil-in-water emulsion. Subsequently, this emulsion was further stirred without lid at 1000 rpm and 37°C for 3 hours to evaporate the cyclohexane and obtain aqueous dispersion of SiNOs. Last, the samples were purified with water three times by centrifuging at 1660*g* and 10°C for 30 min. The SiNO pellets were redispersed in water and kept at 4°C for follow-up experiments.

### Fluorescence labeling of enzymes

Fluorescent dyes Cy3-NHS and Cy5-NHS were dissolved in anhydrous DMSO to prepare stock solutions with a concentration of 10 mg/ml, respectively. Then, 25 μl of Cy3-NHS or Cy5-NHS stock solutions was added dropwise to 1 ml of ADH or ALDH solution (10 mg/ml) and reacted at room temperature in the dark for 4 hours. The labeled enzymes were then purified using 7 kDa molecular weight cutoff (MWCO) Zeba Spin Desalting Columns at 1000*g* for 2 min. The purified enzymes were redispersed in sodium carbonate buffer (100 mM, pH 8.2) for their further encapsulation in SiNOs.

### FRET measurements of fluorescent labeled enzymes in SiNOs

To confirm the close proximity of enzymes in the SiNOs, Cy3-ADH (15 U) and Cy5-ALDH (30 U) were loaded into SiNOs at an active unit ratio of 1:2. For FRET measurement between Cy3 and Cy5, emission spectra of SiNO@Cy3-ADH/Cy5-ALDH were measured under excitation of 500 nm. The mixture of free enzymes Cy3-ADH and Cy5-ALDH at the same ratio as for SiNO@Cy3-ADH/Cy5-ALDH was used for comparison.

### Encapsulation efficiency of enzymes in SiNOs

Encapsulation efficiency of enzymes in SiNOs was determined using the BCA protein assay kit. One milliliter of enzyme-loaded SiNO dispersion was added into a Centrisart ultrafilter tube (MWCO = 300 kDa), followed by ultracentrifugation at 4000*g* for 30 min to obtain the concentrate (Conc.) and the ultrafiltrate (Filtr.). Afterward, the concentration of proteins in the concentrate and ultrafiltrate was measured by mixing the sample and BCA working reagent and incubating for 30 min at 37°C. The absorbance (Abs) at 562 nm was recorded with a microplate reader, and the encapsulation efficiency of enzymes was calculated following the equation $\%\text{EE}={\text{Abs}}_{\text{Conc}.}/\left({\text{Abs}}_{\text{Conc}.}+{\text{Abs}}_{\text{Filtr}.}\right)\times 100\%$.

### Activity evaluation of enzymes

To assess enzyme activity of ADH, native ADH and SiNO@ADH were individually dissolved in a tris-HCl buffer (100 mM, pH 8.0) containing alcohol (0.4% w/v) and NAD^+^ (20 mM). The alcohol degradation reaction was performed at room temperature for 90 min. The generation of acetaldehyde was monitored using the 3-methyl-2-benzothiazolinone hydrazine (MBTH) method at different time points. Briefly, the reaction mixture was equally mixed with MBTH (0.8% w/v) and iron(III) chloride (1% w/v). The formed blue MBTH-acetaldehyde complex was measured at its absorbance maxima at 600 nm. Meanwhile, the absorbance of produced NADH was measured by microplate reader at 340 nm.

For evaluating the enzyme activity of ALDH, native ALDH and SiNO@ALDH were individually dissolved in a tris-HCl buffer (100 mM, pH 8.0) containing KCl (300 mM), 2-mercaptoethanol (50 mM), acetaldehyde (150 μM), and NAD^+^ (20 mM). The absorbance at 340 nm was monitored for tracking the conversion of NAD^+^ to NADH using a microplate reader.

### Tandem photobiocatalysis for alcohol degradation

The NADH/NAD^+^ interconversion and acetaldehyde accumulation in the combined nano-organelles of SiNO@PC and SiNO@ADH/ALDH were determined using the aforementioned methods. The reaction mixture consisted of SiNO@PC (0.5 mg/ml), SiNO@ADH/ALDH (10 mg/ml), alcohol (0.4% w/v), tris-HCl buffer (100 mM, pH 8.0), KCl (300 mM), and NAD^+^ (200 mM). Absorbance intensities of NADH and acetaldehyde (determined using the MBTH method) were recorded using a microplate reader at 340 and 600 nm, respectively, with or without blue light-emitting diode illumination (460 nm). Concentrations of acetaldehyde and NADH were calculated using calibration curves provided in figs. S15 and S16. IPS was calculated using the following equation

$$\text{IPS}=2r\left[{\left(\frac{\;\varphi_{m}}{\;\varphi }\right)}^{1/3}-1\right]\;\left(\text{for}\;\varphi \leq \varphi_{m}\right)$$

where φ_m_ is packing fraction for random dense packing (φ_m_ = 0.63), φ is the particle volume fraction, and *r* is the radius of SiNOs.

### Production of SiNO-encapsulated artificial cells by microfluidics

Chip preparation and microfluidics setup were identical to previous studies (*37*). The inner solution was composed of 100 mM Hepes buffer and 30 vol % SiNOs, while the outer solution consisted of 100 mM sodium chloride in MilliQ water. The middle solution was prepared using a stock solution of block copolymer PB_22_-*b*-PEO_14_ (10 mg/ml) (*M*_n_ = 1200-*b*-600 g mol^−1^) in oleyl alcohol. These solutions were drawn into 1-ml plastic syringes and connected via needles and tubing to the inlets of the microfluidic chip after removing air bubbles within the syringes and tubing. A piece of tubing was connected to the outlet of the chip, which leads the samples to a 1.5-ml Eppendorf tube. The syringes were fixed on the microfluidic pump system, and the chip was monitored with an inverted microscope (Leica DMi 8) equipped with a high-speed camera (Phantom VEO 710, Vision Research). Stable double emulsions were prepared by adjusting the flow rates of outer, middle, and inner fluids. Once the microfluidic chip was successfully initiated, the flow rates were set to 80 μl/hour for the inner and middle phases and 1200 μl/hour for the outer phase. Then, the vesicles were collected via a tube into an external vessel. Following a 15-min period of standing at room temperature for dewetting transition, pGUVs containing SiNOs were obtained for subsequent experiments.

### In vitro biocompatibility assay of synthesized nano-organelles and artificial cells

Approximately 10,000 AML12 (CLS Cell Lines Service GmbH) cells were cultured in each well of a 96-well plate using Dulbecco’s modified Eagle’s medium:F12 complete medium (Gibco) containing 10% fetal bovine serum (FBS), 1% ITS liquid media supplement (100×), dexamethasone (40 ng/ml), and 1% penicillin/streptomycin (P/S). Afterward, the cells were treated with SiNO@PC, SiNO@ADH/ALDH, or pGUV@SiNO (SiNO-loaded pGUVs) at various concentrations (0, 20, 40, 60, 80, 100, 200, and 500 μg/ml) for 24 hours. The biocompatibility of SiNO@PC, SiNO@ADH/ALDH, and pGUV@SiNO was assessed using a cell counting kit-8 (CCK-8). For the CCK-8 assay, 10 μl of CCK-8 solution was added to each well and co-incubated for another 2 hours. Then, the absorbance of each well was measured using a microplate reader at 450 nm, and the viability of AML12 hepatocytes was calculated following the manual of CCK-8 assay kit.

### In vitro alcohol detoxification by hepatocyte-mimicking artificial cells

AML12 cells were seeded overnight in a μ-Slide 8-Well plate (ibidi) with complete medium containing 10% FBS, 1% ITS, dexamethasone (40 ng/ml), and 1% P/S. Subsequently, ArtifCell-A and ArtifCell-B containing 20 mM NAD^+^ were separately cocultured with the cells for 3 hours with 460-nm blue-light irradiation, in the presence of 800 mM alcohol. CLSM was used to monitor intracellular ROS level using fluorescent indicators for oxidative stress assessment. Following artificial cell treatment, the cells were incubated with CellROX Green reagent at 37°C for 30 min. Afterward, additional staining of the plasma membrane was applied for 5 min using CellMask Deep Red. Images of the cell membrane probe (excitation/emission of 649/666 nm) and ROS probe (excitation/emission of 485/520 nm) were captured using CLSM with HCX PL APO CS 63×/1.2 water objective.

### Statistical analysis

All graphs were plotted using GraphPad Prism 9.0 software. All data were calculated and processed as means ± SD of at least three independent experiments. The data were analyzed by two-way analysis of variance (ANOVA) for comparison of trends between different treatments. **P < 0.01 and *P < 0.05 are considered significant differences.
