# Supplementary material for: Integrating incompatible tandem photobiocatalysis in artificial cells enables metabolic modulation of natural cells
Source: Sci Adv. 2025 Jul 4;11(27):eadu4828. doi: 10.1126/sciadv.adu4828 (PMC12227043; doi:10.1126/sciadv.adu4828)
Supplement: Supplementary file 1 — Supplementary Text Figs. S1 to S32 Legend for movie S1 [file sciadv.adu4828_sm.pdf]

Science Advances
AAAS

Supplementary Materials for

Integrating incompatible tandem photobiocatalysis in artificial cells enables metabolic modulation of natural cells

Zhicheng Wang *et al.*

Corresponding author: Shuai Jiang, jiangshuai@ouc.edu.cn; Katharina Landfester, landfest@mpip-mainz.mpg.de

Sci. Adv. **11**, eadu4828 (2025)
DOI: 10.1126/sciadv.adu4828

The PDF file includes:

Supplementary Text
Figs. S1 to S32
Legend for movie S1

Other Supplementary Material for this manuscript includes the following:

Movie S1

Supplementary Text

Materials

2,7-Dibromo-9,9-bis(6-bromohexyl)fluorene, 2,1,3-benzothiadiazole-4,7-diboronic acid pinacol ester, dimethyl sulfone, tetramethyl orthosilicate (TMOS), (3-aminopropyl)triethoxysilane (APTES), acetaldehyde, cell counting kit-8 (CCK-8), propidium iodide (PI), 9,10-anthracenediyl-bis(methylene)dimalonic acid (ABDA), 2,2'-azino-bis(3-ethylbenzothiazoline-6-sulfonic acid) diammonium salt (ABTS), sodium azide, isopropanol (IPA), 4,5-dihydroxy-1,3-benzenedisulfonic acid disodium salt monohydrate (Tiron), Nile Red, $CDCl_3$, CD_2Cl_2, DMSO-d_6, D_2O, horseradish peroxidase (HRP), alcohol dehydrogenase from yeast (ADH), aldehyde dehydrogenase from yeast (ALDH), dexamethasone, and ITS liquid media supplement were purchased from Sigma-Aldrich. Tetrakis(triphenylphosphine)palladium(0), XPhos, diethylamine, iodomethane, 3-methyl-2-benzothiazolone hydrazine (MBTH), BCA protein assay kit, 2,2,6,6-tetramethylpiperidine (TEMP), CellROX green reagent, and CellMask Deep Red plasma membrane stains were obtained from Thermo Fisher. Cy3-NHS ester and Cy5-NHS ester were obtained from Cytiva. β-Nicotinamide adenine dinucleotide (reduced disodium salt, NADH), β-nicotinamide adenine dinucleotide (oxidized, NAD^+), and iron(III) chloride were bought from Carl Roth. Furthermore, Calcein AM (BioLegend), cyclohexane (VWR Chemicals), polyglycerol polyricinoleate (PGPR, GRINDSTED), Lutensol AT50 (BASF), ROS Brite 670 (AAT Bioquest), and poly(butadiene)$_{22}$-*b*-poly(ethylene oxide)$_{14}$ (PB$_{22}$-*b*-PEO$_{14}$, Polymer Source) were used as received. Water with Millipore quality (18.2 megohm·cm) was used in all the experiments.

Characterization methods

^{1}H NMR spectra for photocatalytic polymers were recorded on a Bruker Avance 400 MHz NMR spectrometer. Gel permeation chromatography (GPC, Agilent 1260 Infinity GPC/SEC System), equipped with a PSS SECcurity UV 254 nm detector, was used to determine molecular weights and molecular weight distribution of photocatalytic polymer using HPLC-grade THF as solvent and polystyrene as standard. UV-Vis absorption spectra were measured on a Perkin Elmer Lambda 25 UV-Vis spectrometer and a Tecan Spark microplate reader. Spectra of fluorescence emission and fluorescence resonance energy transfer (FRET) were analyzed by a Tecan Spark microplate reader. The morphology of the silica nanocapsules was examined using a Gemini 1530 (Carl Zeiss, Germany) scanning electron microscope (SEM), Jeol 1400 (Jeol Ltd, Japan) transmission electron microscope (TEM), and Krios G4 (Thermo Scientific, USA) cryo-transmission electron microscope (Cryo-TEM). For the purpose of samples preparation for SEM measurement, 2 μL of silica nanocapsules was dropped onto the silica wafers and dried at room temperature. TEM samples were prepared by depositing a drop of nanocapsules dispersion upon a 300 mesh carbon-coated copper grid and drying at room temperature. To prepare samples for Cryo-TEM measurement, 3 μL of sample was carefully pipetted onto a Quantifoil R 1.2/1.3 300 mesh copper grid, glow discharged in an oxygen plasma cleaner (Diener Nano, Germany). The grid was then immersed in liquid ethane using a Vitrobot Mark IV system and transferred into liquid nitrogen inside the Cryo-TEM for analysis under cryogenic conditions. Fourier transform infrared spectroscopy (FT-IR) measurements were conducted with a Varian 1000 FT-IR spectrometer and analyzed with OPUS software to acquire baseline-corrected absorbance spectra. For each measurement, the potassium bromide (KBr) pellets were produced by blending 2 mg of samples with 300 mg of finely dried KBr. Solid content of nanocapsule dispersions was determined by thermogravimetric analysis (TGA, Mettler Toledo TGA/DSC 3+) with temperature heated up to 500 °C from 25 °C at a rate of 10 K min^{-1} under nitrogen gas. Dynamic light scattering (DLS, Malvern Zetasizer Nano S90) with

a detector at 90° scattering mode was operated to measure average size and size distribution of silica nanocapsules at a temperature of 25 °C. Numbers of polymeric giant unilamellar vesicles (pGUVs) and cells were counted by using Countess II FL Automated Cell Counter (Thermo Scientific). Confocal fluorescence images of pGUVs and cells were captured using a Leica TCS 264 SP5X system.

Calculation for the mass of enzymes and polymers contained within each pGUVs

The inner fluid is composed of 120 μL of SiNOs solution and 280 μL of HEPES buffer. The final concentration of SiNO@PC, SiNO@ADH, or SiNO@ALDH in the pGUVs is 1.35 mg/mL, 8.55 mg/mL, or 17.1 mg/mL, respectively.

To calculate the inner volume of polymersome, it's a hollow sphere with a radius r = 20 μm. The inner volume of one 40 μm polymersome is given by:

$V_{\mathrm{inner}} = 4/3\pi r^3 = 4/3\pi(20\ \mu\mathrm{m})^3 = 3.35\times10^{-5}\ \mu\mathrm{L}$

So, the mass of entrapped SiNO@PC, SiNO@ADH, or SiNO@ALDH in pGUVs is:

$m_{\mathrm{SiNO@PC}} = 3.35\times10^{-5}\ \mu\mathrm{L} \times 1.35\ \mathrm{mg/mL} = 4.52 \times 10^{-5}\ \mu\mathrm{g/polymersome}$

$m_{\mathrm{SiNO@ADH}} = 3.35\times10^{-5}\ \mu\mathrm{L} \times 8.55\ \mathrm{mg/mL} = 28.64 \times 10^{-5}\ \mu\mathrm{g/polymersome}$

$m_{\mathrm{SiNO@ALDH}} = 3.35\times10^{-5}\ \mu\mathrm{L} \times 17.1\ \mathrm{mg/mL} = 57.29 \times 10^{-5}\ \mu\mathrm{g/polymersome}$

When preparation of ADH and ALDH co-encapsulated SiNOs (SiNO@ADH/ALDH), 15 U ADH (0.0375 mg) and 30 U ALDH (12.5 mg) were dissolved in 200 μL of buffer and then loaded into SiNOs. Based on encapsulation efficiency of enzymes in the SiNOs determined by BCA assay, the enzymes were almost completely segregated within the SiNOs (Fig. S18). So, ADH in SiNOs is around 2.08 μg per mg SiNOs and ALDH in SiNOs is around 138.89 μg per mg SiNOs. From the TGA data of SiNO@PC (Fig. S9), PC in SiNOs is around 20.11 μg per mg SiNOs.

The average mass of PC, ADH, and ALDH in each pGUVs is:

$m_{\mathrm{PC}} = 4.52 \times 10^{-11}\ \mu\mathrm{g/polymersome} \times 20.11\ \mu\mathrm{g/mg_{SiNOs}} = 9.09 \times 10^{-4}\ \mu\mathrm{g/polymersome}$

$m_{\mathrm{ADH}} = 28.64 \times 10^{-11}\ \mu\mathrm{g/polymersome} \times 2.08\ \mu\mathrm{g/mg_{SiNOs}} = 5.96 \times 10^{-4}\ \mu\mathrm{g/polymersome}$

$m_{\mathrm{ALDH}} = 57.29 \times 10^{-11}\ \mu\mathrm{g/polymersome} \times 138.89\ \mu\mathrm{g/mg_{SiNOs}} = 7.96 \times 10^{-2}\ \mu\mathrm{g/polymersome}$

Hence, the average PC, ADH, and ALDH in each pGUVs is approximately 9.09 × 10^{-4} μg, 5.96 × 10^{-4} μg, and 7.96 × 10^{-2} μg, respectively.

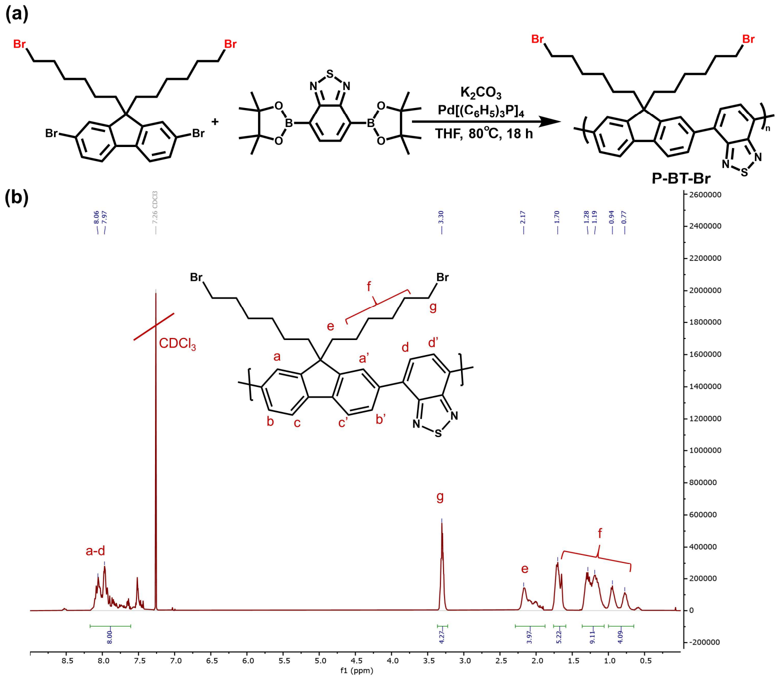

Fig. S1. P-BT-Br synthesis. (a) Synthetic route of P-BT-Br and (b) ^{1}H NMR spectrum of P-BT-Br in $CDCl_3$ (400 MHz).

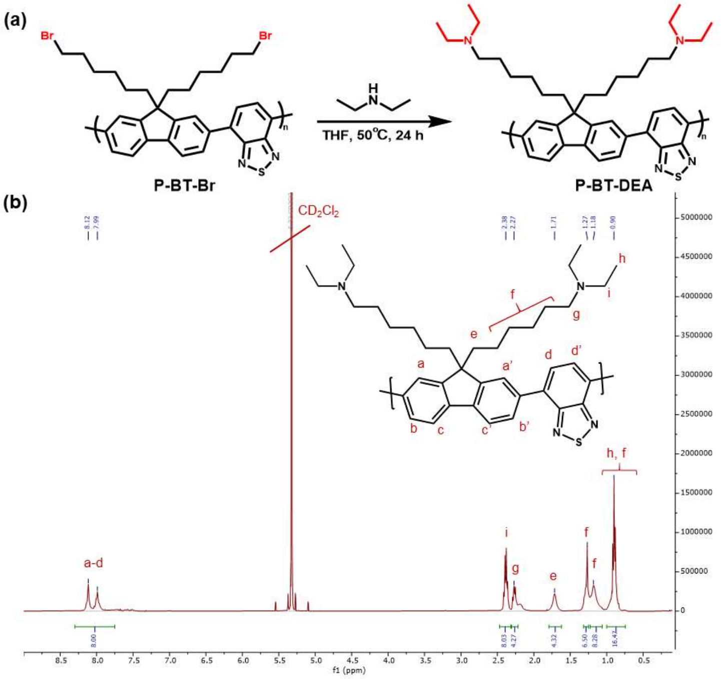

Fig. S2. P-BT-DEA synthesis. (a) Synthetic route of P-BT-DEA and (b) ^{1}H NMR spectrum of P-BT-DEA in CD_2Cl_2 (400 MHz).

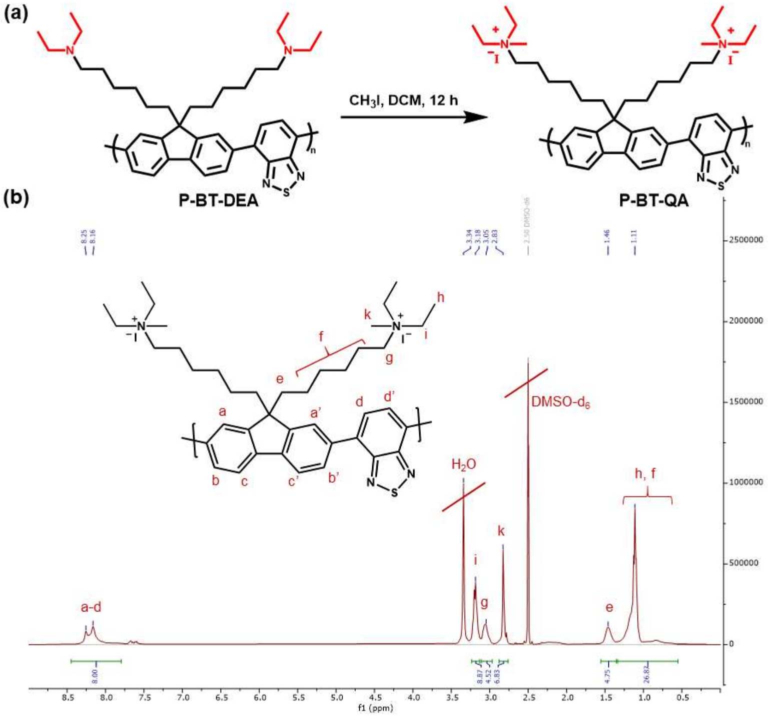

Fig. S3. P-BT-QA synthesis. (a) Synthetic route of P-BT-QA and (b) ^{1}H NMR spectrum of P-BT-QA in DMSO-d_6 (400 MHz).

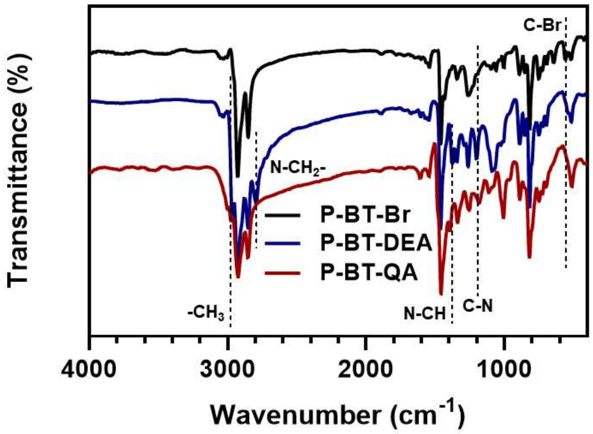

Fig. S4. FT-IR analysis of photocatalytic polymers P-BT-Br, P-BT-DEA, and P-BT-QA.

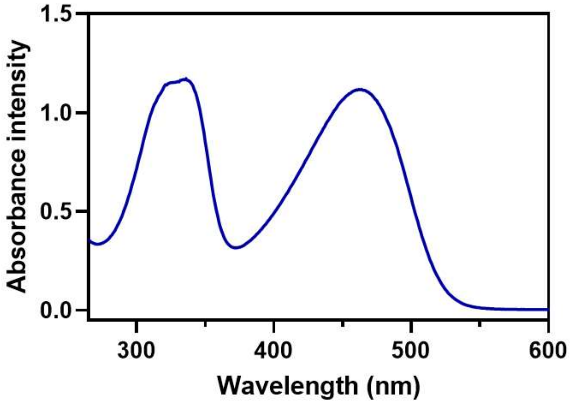

Fig. S5. UV-Vis absorption curve of P-BT-QA photocatalytic polymer (PC).

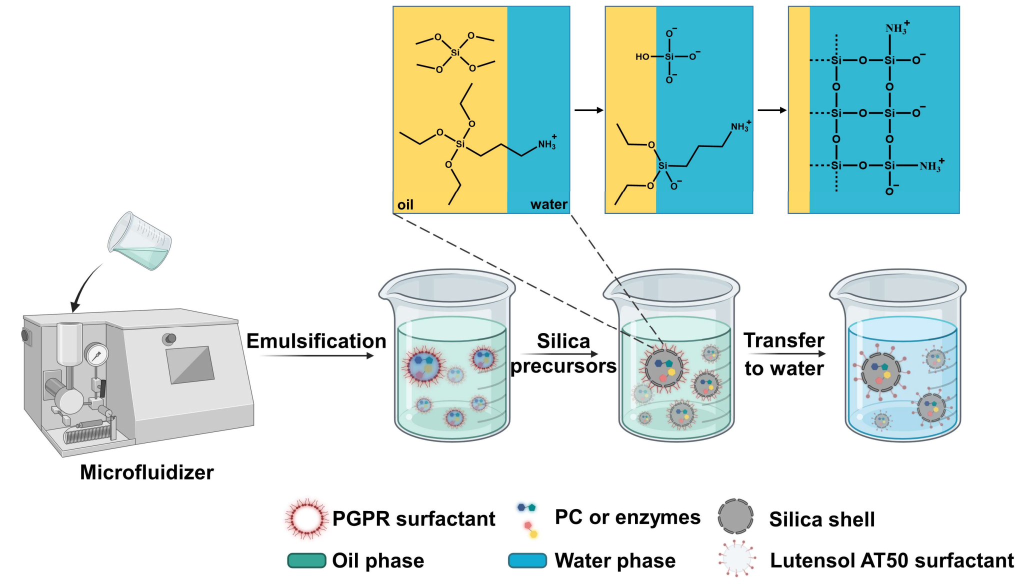

Fig. S6. *In situ* encapsulation of photocatalytic polymers or enzymes in silica nano-organelles by inverse miniemulsion polymerization. The oil phase and water phase were mixed and processed by a microfluidizer for emulsification. Subsequently, silica precursors consisting of TMOS and APTES were added dropwise to the resulting emulsion. Amino group of APTES is protonated at water-oil interface, resulting in amphiphilic $APTES^{+}$. Methoxy groups of TMOS and ethoxy groups of $APTES^{+}$ are hydrolyzed to hydrophilic silanol groups (Si-OH). The silanol groups are deprotonated to $Si\text{-}O^{-}$ at pH above the isoelectric point (pH 2-3). Hence, silica shell is formed at water-oil interface due to electrostatic interactions between $APTES^{+}$ and $Si\text{-}O^{-}$, from co-condensation of silanol groups from APTES and TMOS. Finally, the formed silica nano-organelles in oil phase are transferred to an aqueous phase with the aid of Lutensol AT50 as surfactant. *The figure was created with BioRender.com.*

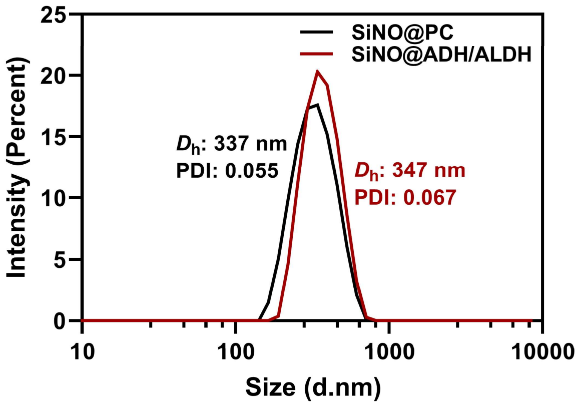

Fig. S7. Size distribution of SiNO@PC and SiNO@ADH/ALDH measured by DLS.

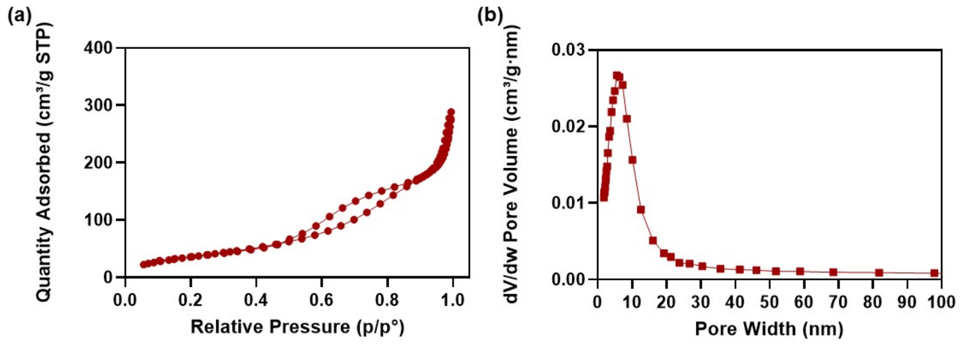

Fig. S8. BET analysis. (a) N_2 adsorption-desorption isotherms and (b) pore size distribution curve of the silica nano-organelles.

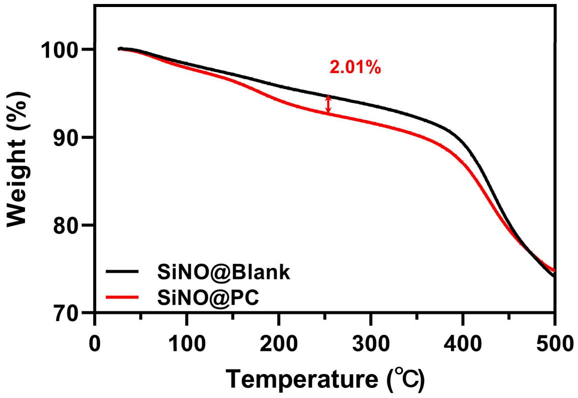

Fig. S9. Thermogravimetric analysis (TGA) of SiNO@Blank and SiNO@PC.

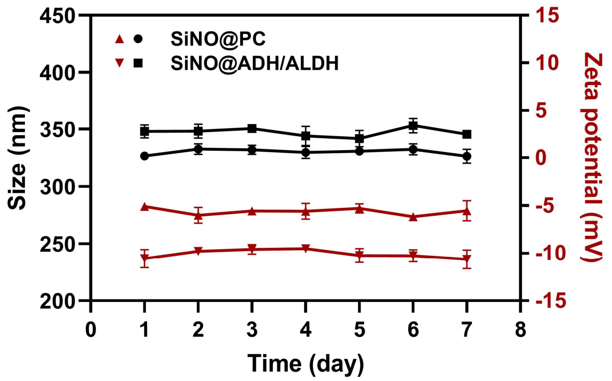

Fig. S10. Size and zeta potential stability test of SiNO@PC and SiNO@ADH/ALDH over a period of 7 days. Data are presented as the mean ± SD for n = 3 independent samples.

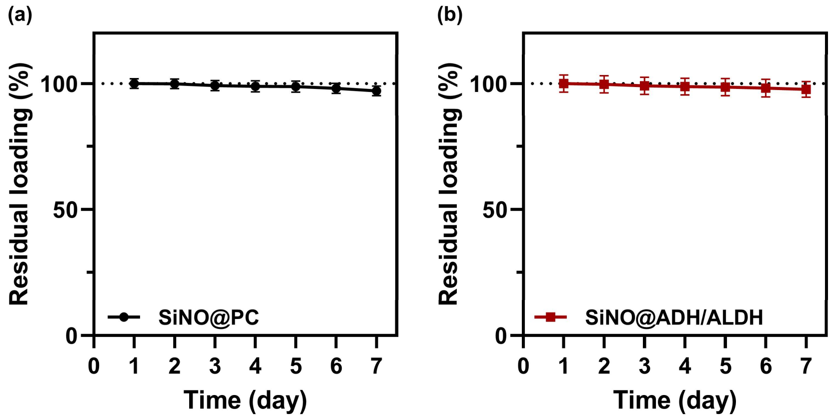

Fig. S11. Residual loading in SiNOs. Residual loading amount of PC and ADH/ALDH in (a) SiNO@PC and (b) SiNO@ADH/ALDH over a period of 7 days. Data are presented as the mean ± SD for n = 3 independent samples.

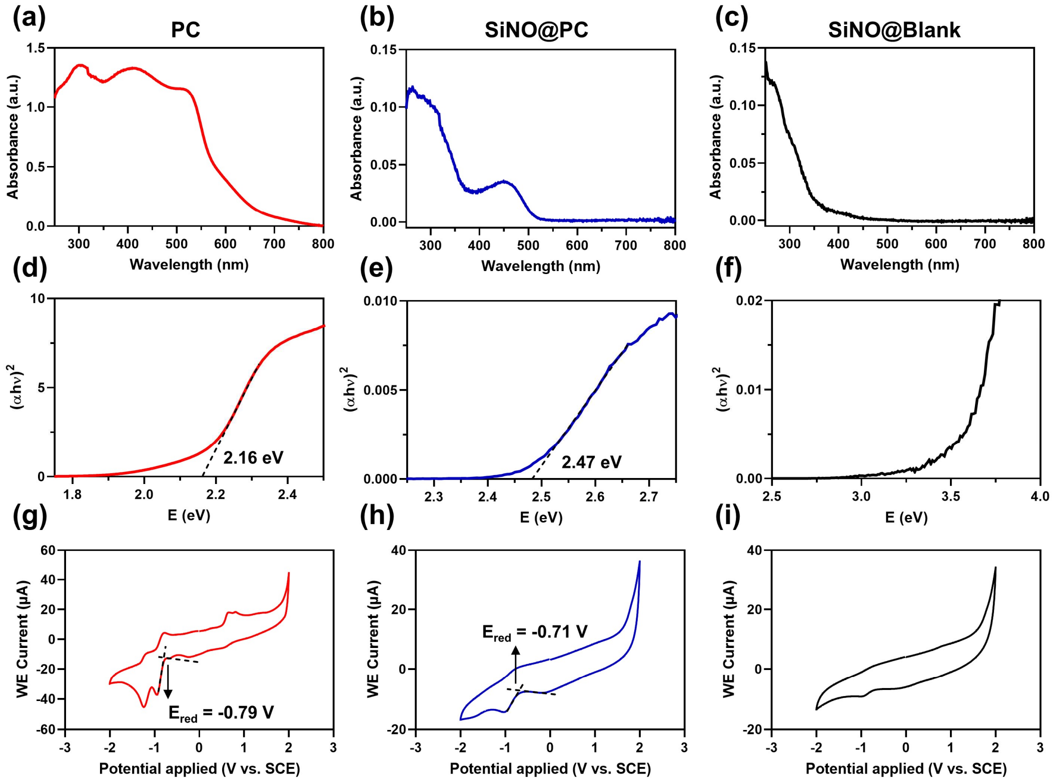

Fig. S12. Optical and electronic properties characterization. UV-Vis DRS of (a) PC, (b) SiNO@PC, and (c) SiNO@Blank. Kubelka-Munk transformed UV-Vis reflectance spectra of (d) PC, (e) SiNO@PC, and (f) SiNO@Blank. Cyclic voltammetry of (g) PC, (h) SiNO@PC, and (i) SiNO@Blank.

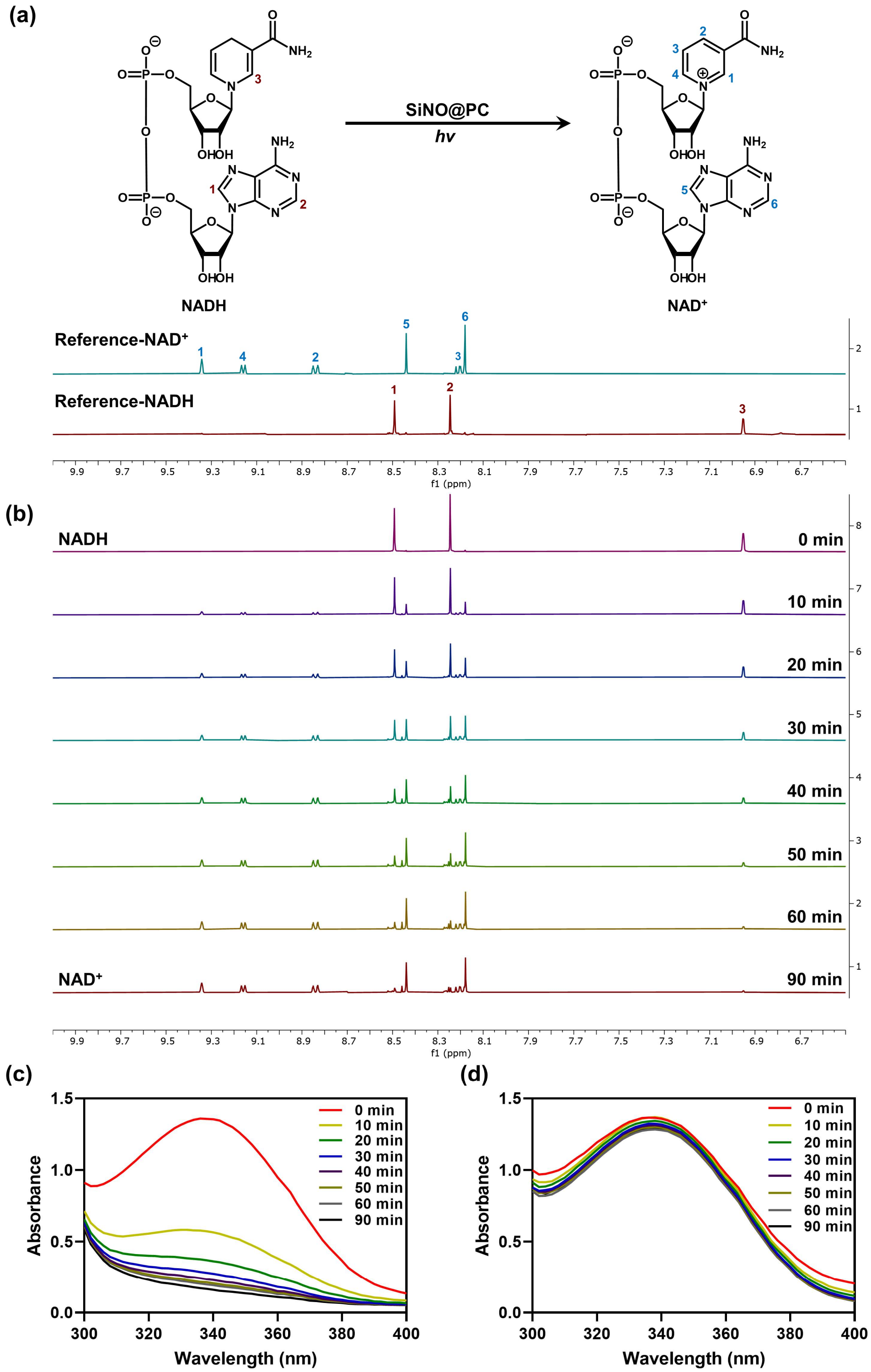

Fig. S13. Photocatalytic oxidation of NADH to NAD^+ by SiNO@PC. (a) Reaction equation and ^1H NMR spectra of standard references. (b) Change in ^1H NMR spectra of NADH upon oxidation by SiNO@PC at different irradiation times (λ = 460 nm, 1 mM NADH in D_2O). Absorbance spectra of NADH at different irradiation times with SiNO@PC (c) and SiNO@Blank (d).

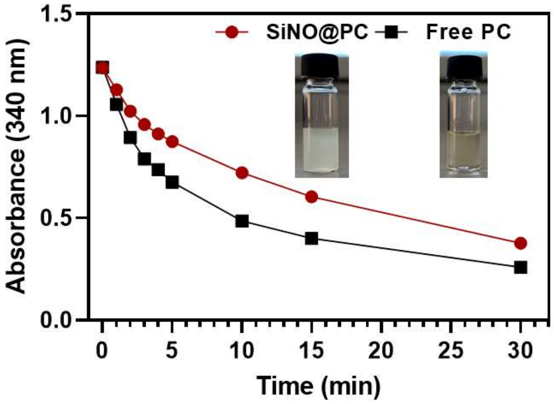

Fig. S14. Comparison of SiNO@PC and free PC in NADH oxidation. Changes in the absorbance of NADH (1 mM) catalyzed by SiNO@PC (containing 5 μg/mL PC) or free PC (5 μg/mL) under light irradiation (10 mW/cm^2, λ = 460 nm).

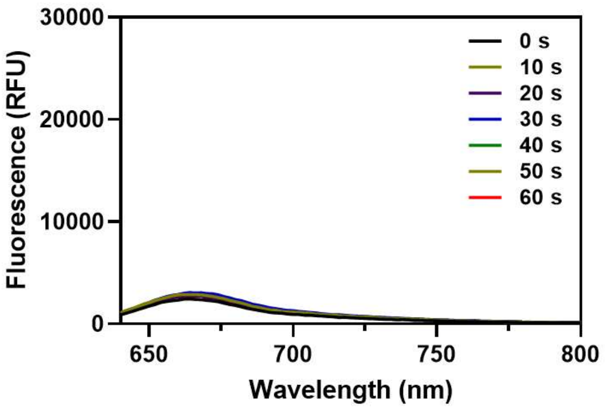

Fig. S15. Fluorescence spectra of ROS Brite 670. Change in fluorescence spectra of ROS Brite 670 (λ_{ex} = 600 nm) upon treatment with SiNO@Blank under light irradiation over a period of 60 s. The ROS Brite 670 is a ROS-responsive probe that can be excited from non-fluorescence to a fluorescent state (λ_{em} = 670 nm) by ROS oxidation.

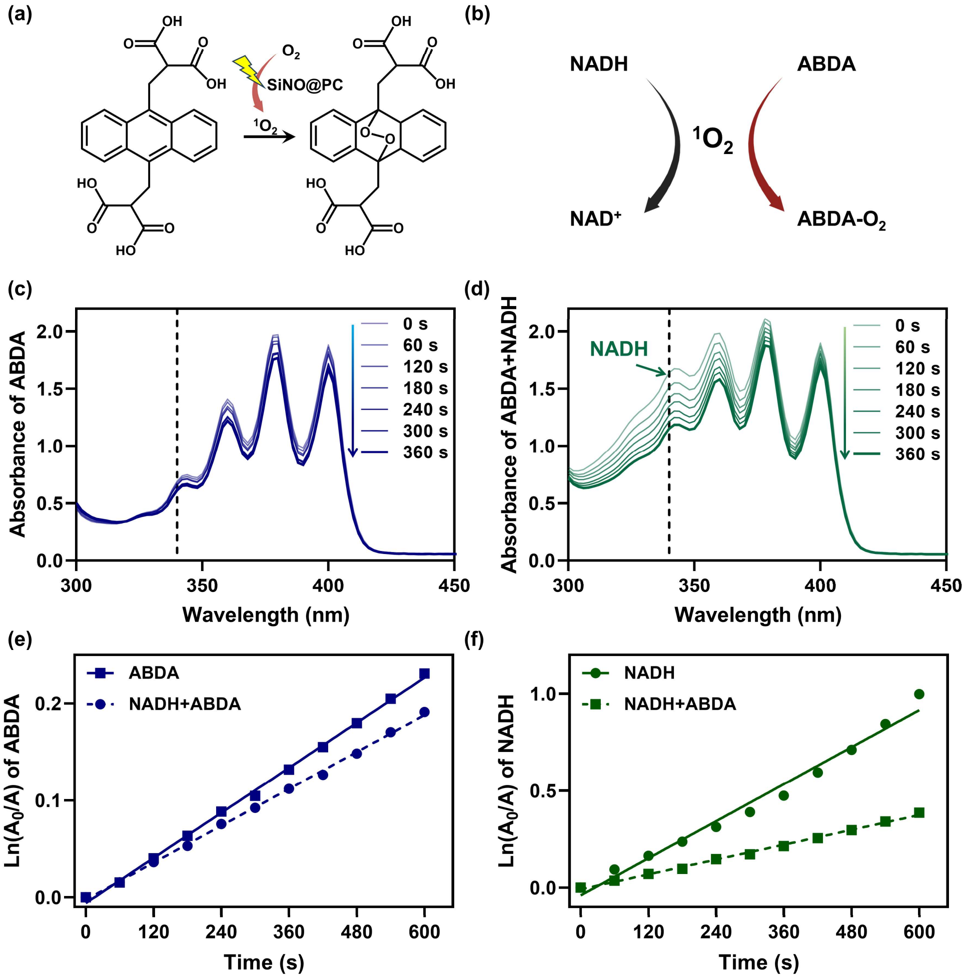

Fig. S16. Competitive reaction of $^1O_2$ between 9,10-anthracenediyl-bis(methylene)dimalonic acid (ABDA) and NADH. (a) Mechanism of ABDA as a scavenger for the detection of $^1O_2$ in solution. (b) Schematic illustration of the competitive reaction between ABDA and NADH involving singlet oxygen. The absorption spectra of ABDA (1 mM) after irradiation with blue light (5 mW/cm^2, λ = 460 nm) in the presence of SiNO@PC for different time periods without NADH (c) or with NADH (d). (e) Plots of ln(A_0/A) for ABDA absorbance at 400 nm in the absence of NADH (ABDA) and in the presence of NADH (NADH+ABDA). (f) Plots of ln(A_0/A) for NADH absorbance at 340 nm in the absence of ABDA (NADH) and in the presence of ABDA (NADH+ABDA). A_0 and A represent the absorbance of ABDA at 400 nm or NADH at 340 nm, prior to (t = 0) and after (t) irradiation, respectively. The slope of the linear fit indicates the oxidative rate of ABDA or NADH.

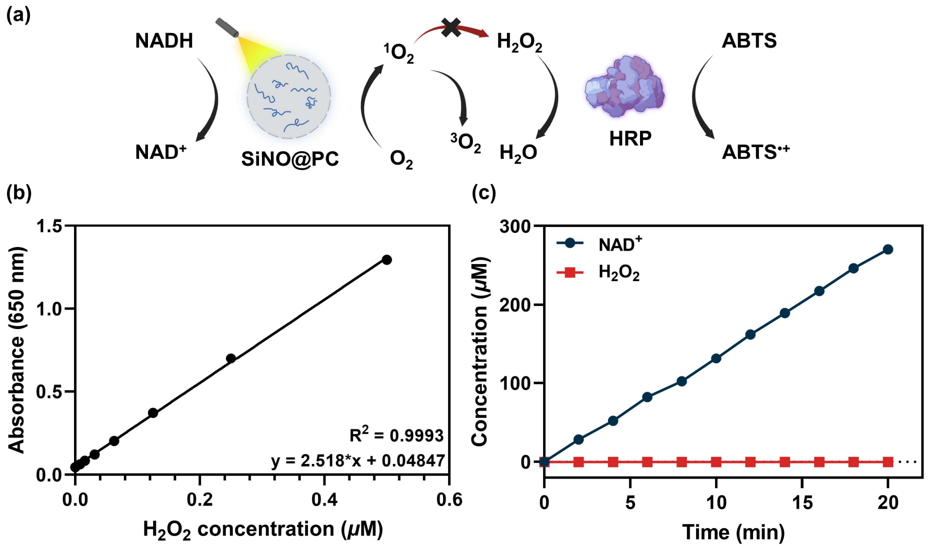

Fig. S17. Product measurement of SiNO@PC under light irradiation. (a) Schematic representation of the measurement of hydrogen peroxide. During NAD^+ regeneration catalyzed by SiNO@PC, generated $^1O_2$ either forms H_2O_2 or reverts to ground state oxygen ($^3O_2$). Only H_2O_2 is further involved in HRP-coupled ABTS oxidation reaction. (b) Standard curve of H_2O_2 measured by $ABTS^{\bullet+}$ absorbance at 650 nm. The oxidation reaction of ABTS (colorless) to $ABTS^{\bullet+}$ (blue, absorbance at 650 nm) was catalyzed by HRP in the presence of different concentrations of H_2O_2. (c) Generation of NAD^+ and H_2O_2 by SiNO@PC under light irradiation (10 mW/cm², λ = 460 nm) for different time periods. Data are presented as the mean ± SD for n = 3 independent samples.

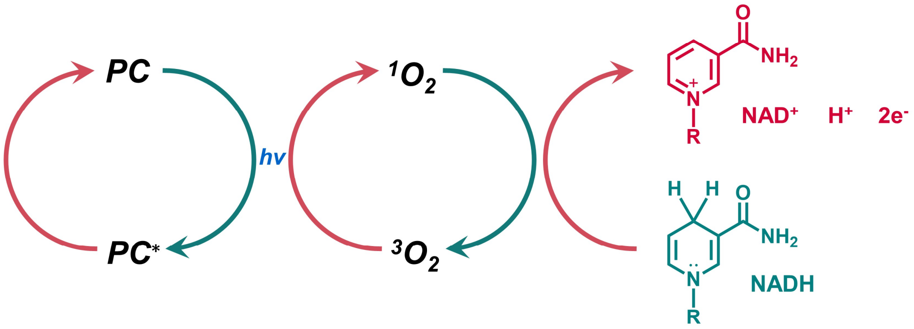

Fig. S18. The proposed mechanism of photocatalytic polymer-mediated NAD^+ regeneration. The proposed mechanism involves the following reactions: $PC + hv \rightarrow PC^*(h^+ + e^-)$; $PC^* + {}^3O_2 \rightarrow PC^* + {}^1O_2$; $NADH + {}^1O_2 \rightarrow NAD^+ + O_2 + H^+ + 2e^-$.

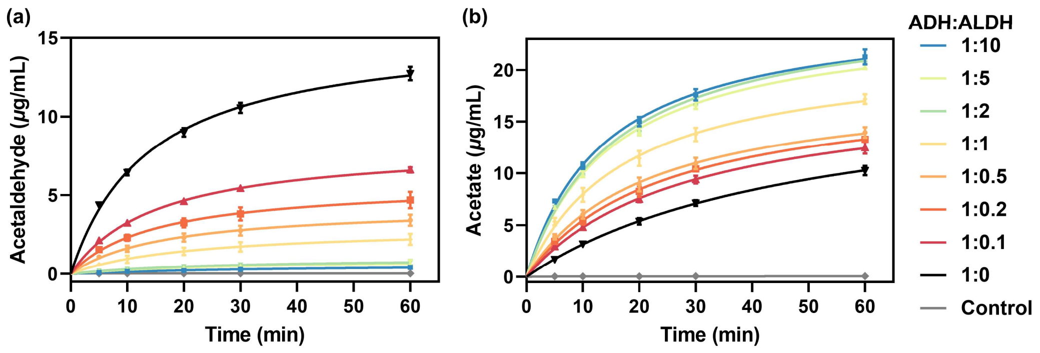

Fig. S19. Comparison of acetaldehyde accumulation and acetate production at different ADH/ALDH ratios. (a) The accumulation of acetaldehyde and (b) the production of acetate were measured over different time periods.

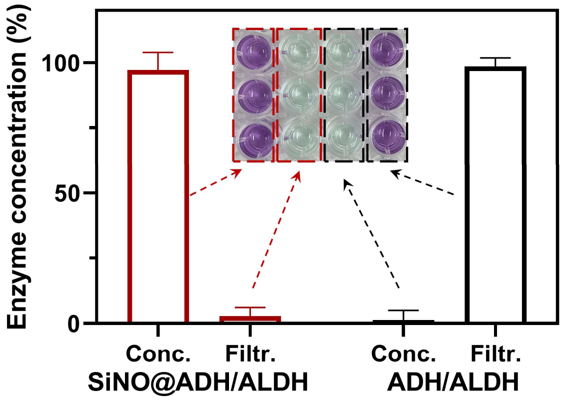

Fig. S20. Encapsulation efficiency of enzymes in the SiNOs determined by BCA assay. Firstly, the dispersion of SiNO@ADH/ALDH was ultrafiltrated (MWCO = 300 kDa) in order to separate the SiNO from non-encapsulated free enzymes. The obtained concentrate (Conc.) and ultrafiltrate (Filtr.) were measured with BCA assay. To prove the permeability of ultrafiltration membrane for the free enzymes ADH (Mw 141-151 kDa) and ALDH (Mw 228 kDa), a mixed solution of ADH and ALDH (non-encapsulated form) was ultracentrifuged as a control experiment. The proteins react with BCA working solution to generate purple product.

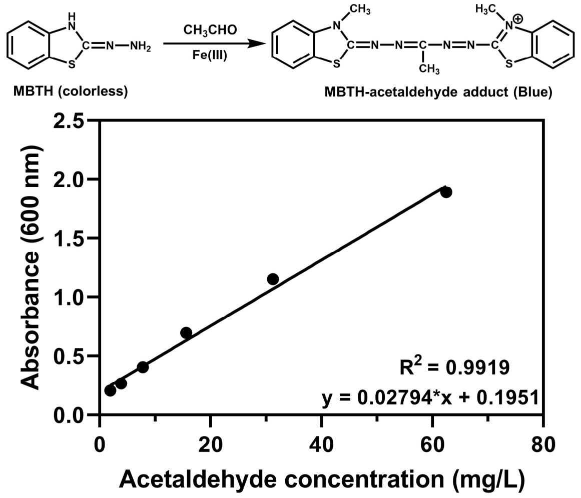

Fig. S21. MBTH method for acetaldehyde detection. Reaction equation of MBTH method for the detection of acetaldehyde and the standard curve of acetaldehyde absorbance at 600 nm.

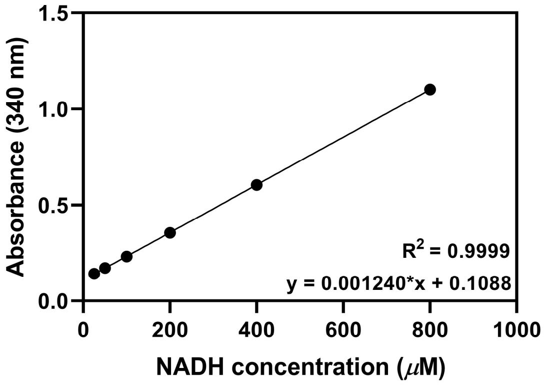

Fig. S22. Standard curve of NADH absorbance at 340 nm.

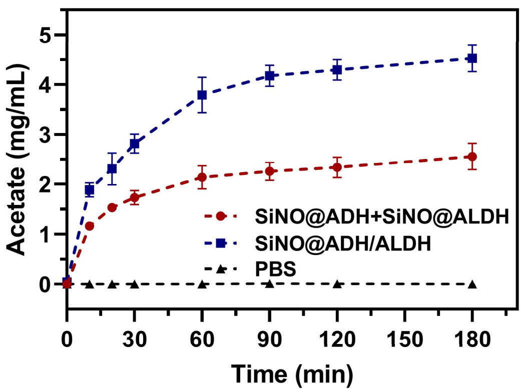

Fig. S23. Concentration changes of acetate. Concentration of acetate following incubation with SiNO@ADH+SiNO@ALDH or SiNO@ADH/ALDH. Data are presented as the mean ± SD for n = 3 independent samples.

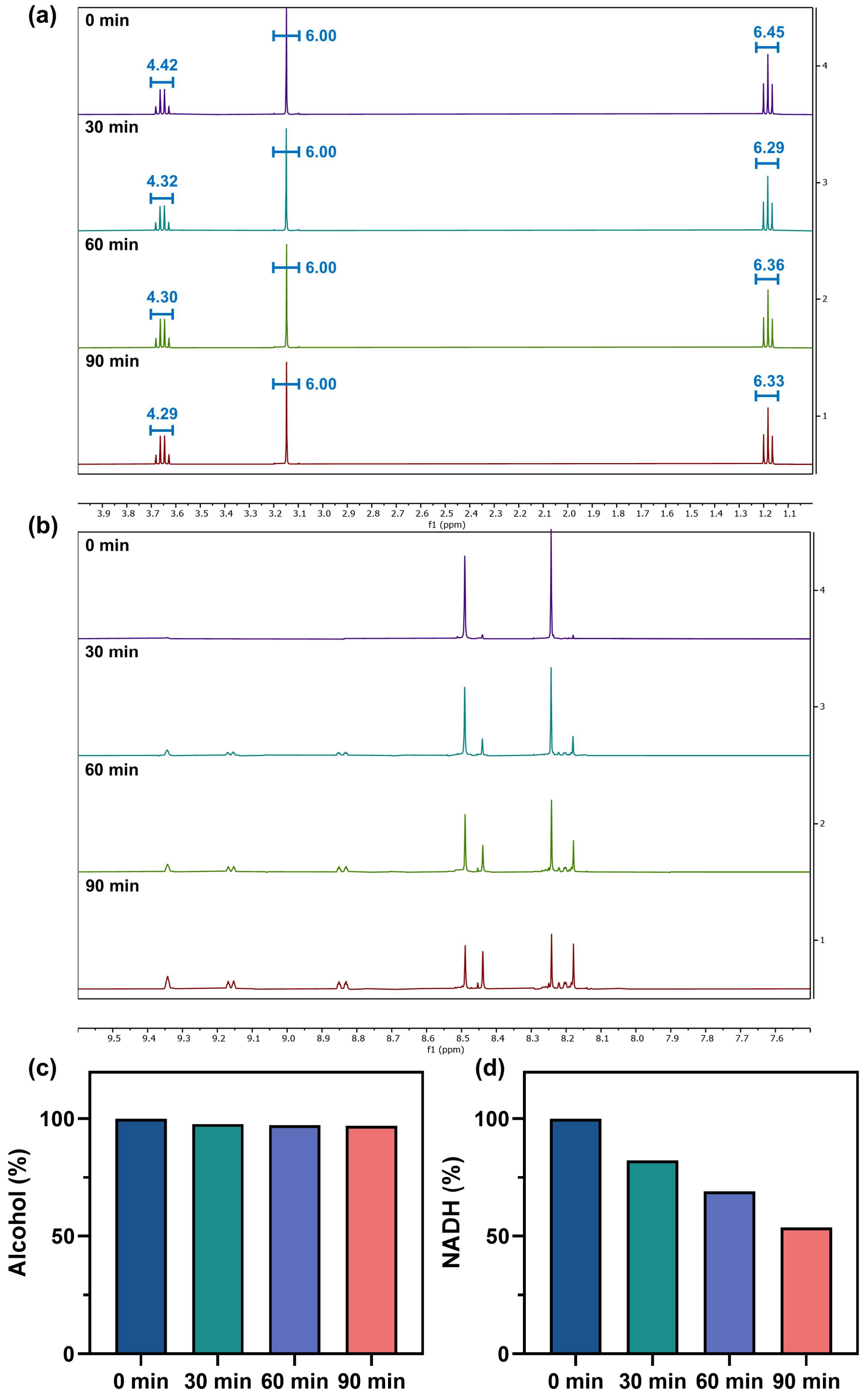

Fig. S24. Quantitative ^{1}H NMR spectra. Quantitative ^{1}H NMR spectra of alcohol (a) and NADH (b) upon catalysis by SiNO@PC at different irradiation times (λ = 460 nm). Residual percentages of alcohol (c) and NADH (d) after different irradiation times. Dimethyl sulfone (10 mg/mL in D_2O) was used as an internal standard.

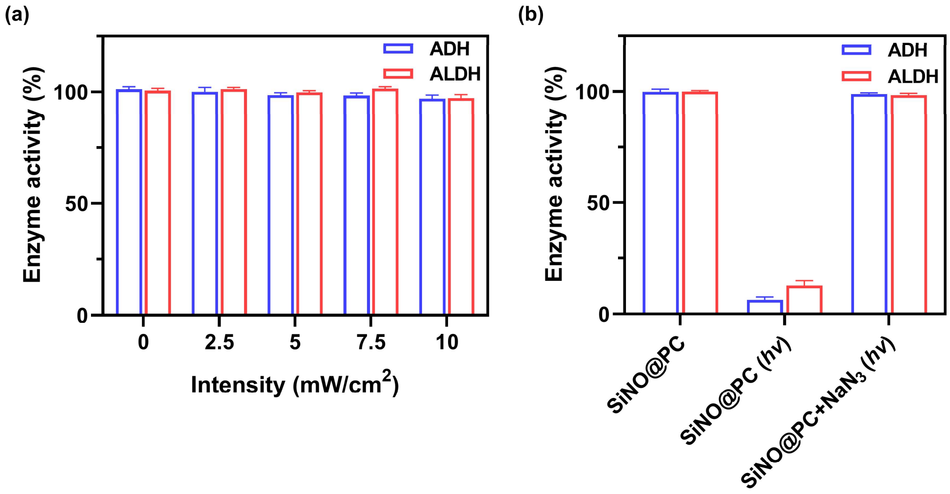

Fig. S25. Relative enzyme activity analysis. (a) Relative enzyme activity of ADH and ALDH following treatment with 460 nm blue light at various intensities. (b) Relative enzyme activity of ADH and ALDH after mixing with SiNO@PC under different conditions for 1 h. SiNO@PC, SiNO@PC (*hv*), and SiNO@PC + NaN_3 (*hv*) represent the mixture of SiNO@PC with enzyme, SiNO@PC with enzyme under light irradiation, and SiNO@PC with enzyme and NaN_3 under light irradiation, respectively. *hv* = 460 nm blue light, 10 mW/cm^2. Sodium azide (NaN_3) was introduced as a scavenger for quenching PC-generated singlet oxygen. Data are presented as the mean ± SD for n = 3 independent samples.

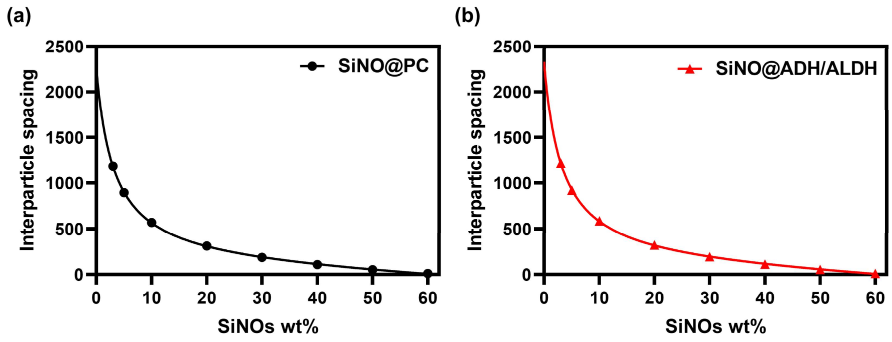

Fig. S26. Plots of calculated average interparticle spacing. Average interparticle spacing of (a) SiNO@PC and (b) SiNO@ADH/ALDH as a function of weight percentage in solution (wt%).

(a)

Outer fluid	NaCl (100 mM)
Middle fluid	PB-PEO (10 mg/mL) in oleyl alcohol
Inner fluid	•SiNO@PC (4.3 mg/mL)<br>•SiNO@ADH/ALDH or SiNO@ADH+SiNO@ALDH (85.8 mg/mL)<br>•HEPES (100 mM)

(b)

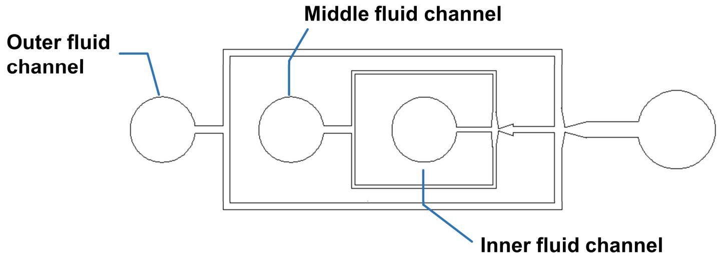

Fig. S27. Device of microfluidic chip. (a) Composition of the fluids for the production of pGUVs-based artificial cells by microfluidics. (b) Chip construction for artificial cell preparation.

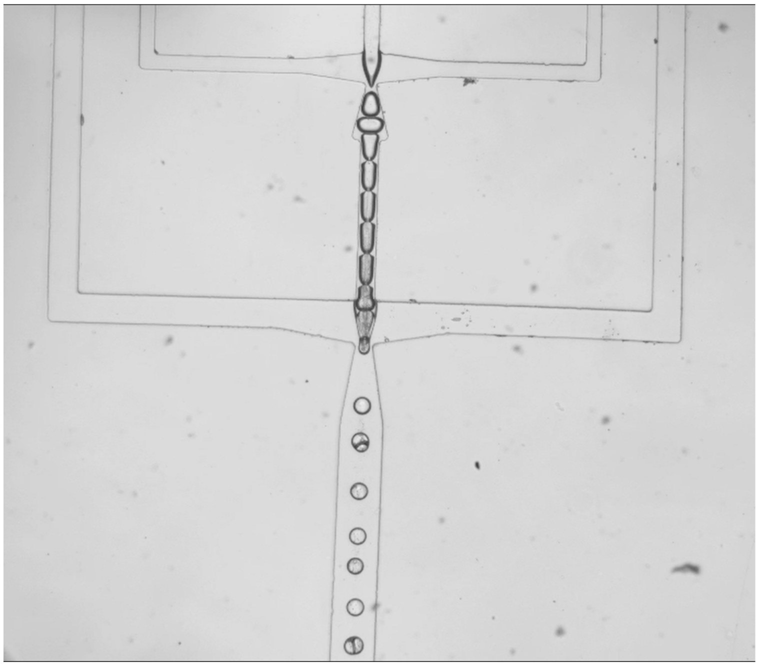

Fig. S28. Bright-field image of preparation of silica nano-organelles loaded pGUVs.

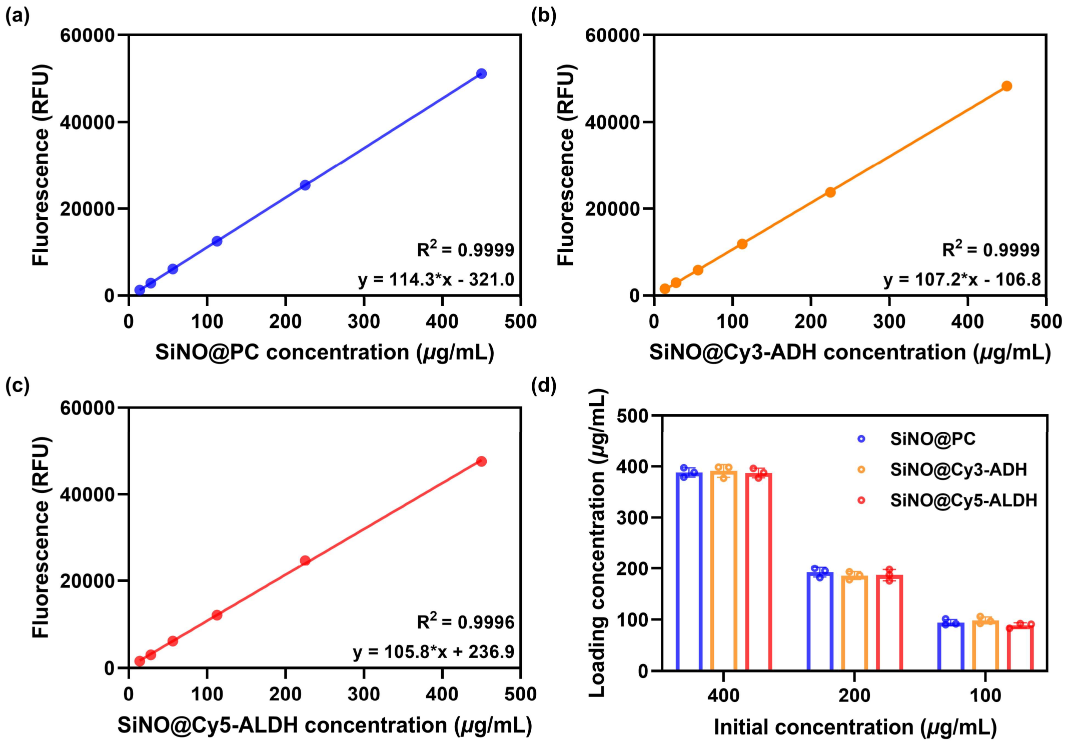

Fig. S29. Quantitative analysis of loaded SiNOs in pGUVs. Standard fluorescence intensity curves for (a) SiNO@PC, (b) SiNO@Cy3-ADH, and (c) SiNO@Cy5-ALDH. (d) Quantitative analysis of loading concentrations of SiNO@PC, SiNO@Cy3-ADH, and SiNO@Cy5-ALDH in pGUVs at varied initial concentrations but with identical feeding mass ratio of 1:1:1. After co-encapsulation, fluorescence intensities of each SiNOs were measured using a microplate reader, and the corresponding loading concentrations were calculated based on their standard fluorescence intensity curves.

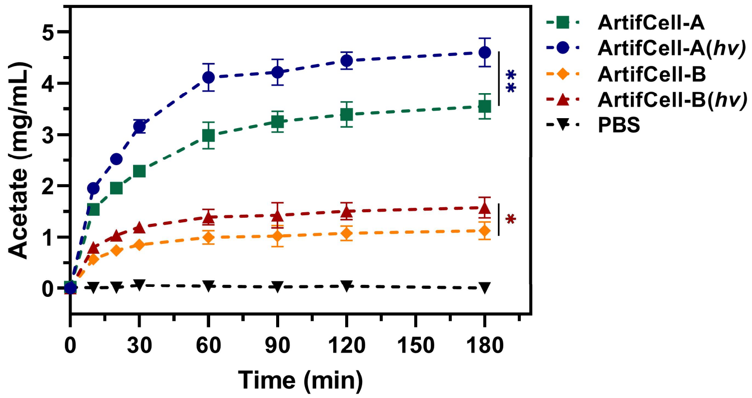

Fig. S30. Concentration changes of acetate. Concentration of acetate following incubation with ArtifCell-A, ArtifCell-A(*hv*), ArtifCell-B, ArtifCell-B, or ArtifCell-B(*hv*); *hv* = 460 nm blue light, 5 mW/cm^2. The data are presented as the mean ± SD for n = 3 independent samples. *$P < 0.05$; **$P < 0.01$.

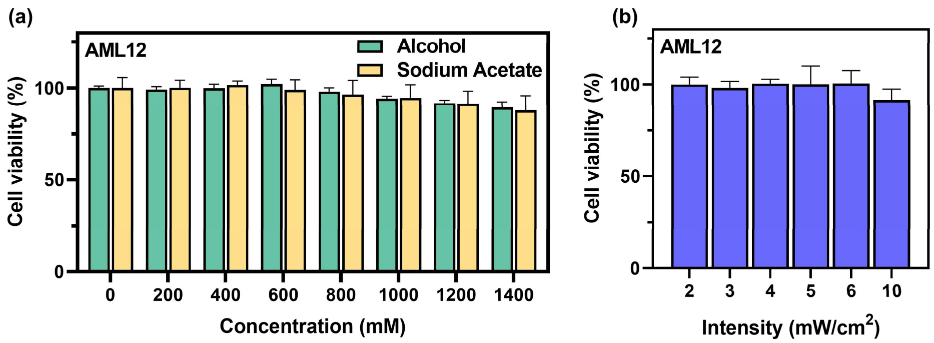

Fig. S31. Cell viability measurement. Viability of AML12 hepatocytes following incubation with alcohol and sodium acetate at various concentrations (a) or irradiation with blue light at various intensities (b) for 3 h. Data are presented as the mean ± SD for n = 3 independent samples.

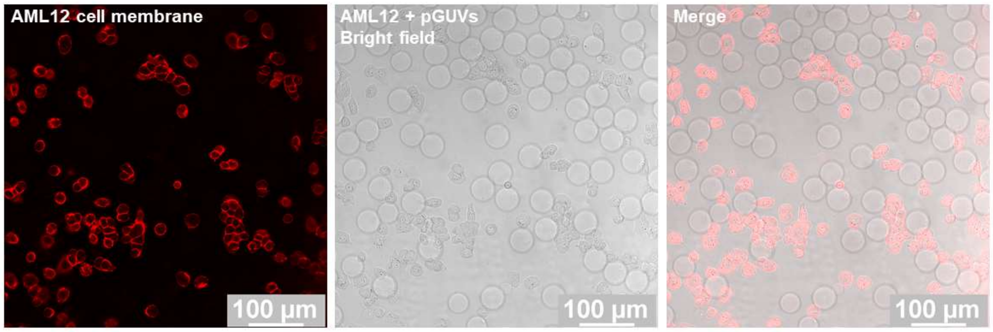

Fig. S32. CLSM images of co-cultured AML12 cells and pGUVs after 3 h. Cell membrane was stained with CellMask Deep Red.

Movie S1. Preparation of pGUVs-based artificial cells using microfluidics.
